# Exploring the Association of Electron‐Donating Corroles with Phthalocyanines as Electron Acceptors

**DOI:** 10.1002/chem.202103891

**Published:** 2022-02-10

**Authors:** Benedikt Platzer, Beatrice Berionni Berna, Martina Bischetti, Daniel O. Cicero, Roberto Paolesse, Sara Nardis, Tomás Torres, Dirk M. Guldi

**Affiliations:** ^1^ Department of Chemistry and Pharmacy Interdisciplinary Center for Molecular Materials (ICMM) Friedrich-Alexander-Universität Erlangen-Nürnberg Egerlandstr. 3 91058 Erlangen Germany; ^2^ Department of Chemical Science and Technologies University of Rome Tor Vergata Via della Ricerca Scientifica 00133 Rome Italy; ^3^ Departamento de Química Orgánica Universidad Autónoma de Madrid, Campus de Cantoblanco C/ Francisco Tomás y Valiente 7 28049 Madrid Spain; ^4^ Institute for Advanced Research in Chemical Sciences (IAdChem) Universidad Autónoma de Madrid, Campus de Cantoblanco 28049 Madrid Spain; ^5^ IMDEA-Nanociencia C/Faraday 9, Campus de Cantoblanco 28049 Madrid Spain

**Keywords:** electron transfer, organic photovoltaics, porphyrinoids, supramolecular chemistry

## Abstract

Electron‐donating corroles (Cor) were integrated with electron‐accepting phthalocyanines (Pc) to afford two different non‐covalent Cor ⋅ Pc systems. At the forefront was the coordination between a 10‐*meso*‐pyridine Cor and a ZnPc. The complexation was corroborated in a combination of NMR, absorption, and fluorescence assays, and revealed association with binding constants as high as 10^6^ m ^−1^. Steady‐state and time‐resolved spectroscopies evidenced that regardless of exciting Cor or Pc, the charge‐separated state evolved efficiently in both cases, followed by a slow charge‐recombination to reinstate the ground state. The introduction of non‐covalent linkages between Cor and Pc induces sizeable differences in the context of light harvesting and transfer of charges when compared with covalently linked Cor‐Pc conjugates.

## Graphical abstract

**Non‐covalent corrole‐(Zn)phthalocyanine** conjugates were afforded by mixing electron‐donating 10‐*meso*‐pyridine corroles with an electron‐accepting phthalocyanine. Investigation of the complexation by NMR, absorption, and fluorescence essays revealed binding constants as high as 10^6^ m ^−1^. Introducing non‐covalent corrole‐phthalocyanine linkages induces sizeable differences in the context of light harvesting and charge‐transfer when compared to covalently linked equivalents.

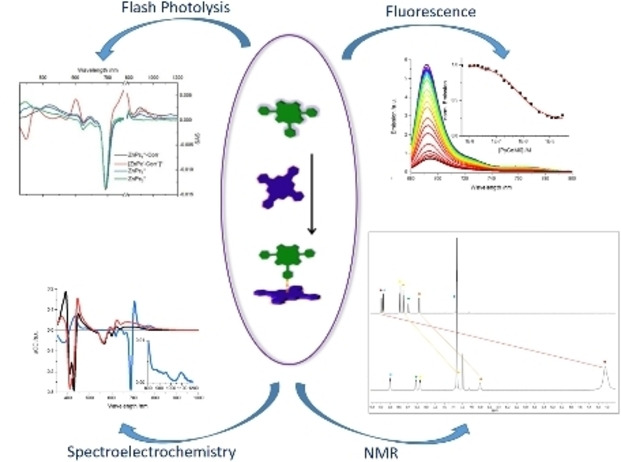

## Introduction

Photoinduced electron‐ and energy‐transfer processes play a primary role in natural photosynthesis, which constitutes nature's way of converting light into usable chemical energy.^[1]^ More specifically, sunlight excites the natural antenna chromophores to provide electronic excitation, which is funneled to the reaction center by a cascade of electron and energy transfer processes.^[2]^ The success of this process relies on the effectiveness of these electron transfers and the lack of recombination reactions that would interrupt the process and cause a waste of the absorbed energy.^[3]^ The fine structure‐function‐reactivity relationship that exists for naturally occurring photosynthetic reaction centers has prompted the design and the preparation of a variety of electron donor‐acceptor ensembles, which have been developed and applied to many technologically relevant research fields, such as light‐electricity conversion, light‐fuel production, and optoelectronic devices.^[4]^

Chlorophyll and bacteriochlorophylls, the most common of visible‐light absorbing building blocks found in nature, are only one type of a more general subset of biologically inspired pigments composed, at their cores, of tetrapyrrolic macrocycles. Thus, porphyrinoids represent the natural choice in the design of multicomponent electron‐donor‐acceptor systems to mimic the natural reaction center as a replacement of the synthetically demanding and relatively unstable chlorophylls.^[5]^

Corroles (Cor) and likewise phthalocyanines (Pc), have been extensively studied owing to their roles in mimicking biological systems.^[6]^ Their high thermal and chemical stability, their optical and redox properties,^[7]^ their tunability of these features by complexation with different metals or by different substitution at their peripheries render them excellent candidates for this purpose. Cor, with their maximum absorption in the range of 400–450 nm,^[8]^ together with the Pc maximum absorption centered at 600–700 nm,^[9]^ cover a large range of the solar spectrum, that is, from the UV to the near IR. Cor have widely been incorporated into multicomponent conjugates as electron donors due to their low oxidation potential, especially when compared with the porphyrin analogues.^[10]^ Pc have been proven to act as excellent electron acceptor counterparts, particularly when endowed with strong electron‐withdrawing substituents.^[11]^

Very recently, two conjugated panchromatic Cor‐Pc conjugates have been reported.^[12]^ In both cases, the two differently substituted Cor operate as primary electron donors after photoexcitation, while the conjugated electron accepting Pc functions as primary electron acceptor.

It is noteworthy that in the natural systems the light harvesters of the antennas as well as the electron donors and electron acceptors in the reaction centers are brought together through non‐covalent interactions. Compared with artificial light‐transforming systems, in which covalent linkages are employed, light‐harvesting systems based on non‐covalent interactions undoubtedly present several benefits. First, they are easier to fabricate without the needs of multiple synthetic steps. Second, the solution stability is tunable by the careful choice of the supramolecular interactions and/or the external stimuli. Third, the control over the assembly/disassembly of such supramolecular systems does allow perturbing and modulating some of their physicochemical properties. Among the many different non‐covalent interactions, metal‐ligand coordination is an efficient means to self‐assemble Cor‐ and Pc‐based electron donor‐acceptor ensembles.^[13]^

**Scheme 1:**
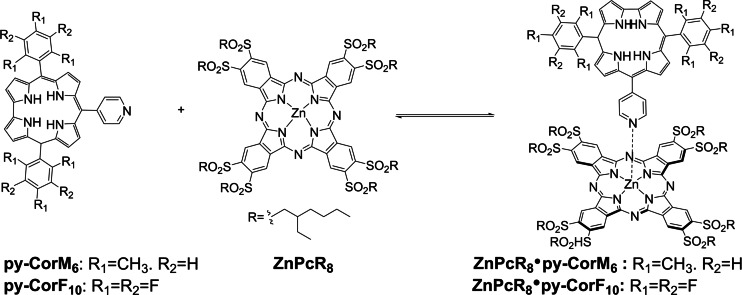
Metal‐ligand, axial coordination of either **py‐CorM_6_** or **py‐CorF_10_** with **ZnPcR_8_** to afford **ZnPcR_8_** ⋅ **py‐CorM_6_** and **ZnPcR_8_** ⋅ **py‐CorF_10_**.

In the current study, two different Cor, bearing substituents with drastically different electronic behaviour in their 5 and 15 *meso*‐positions were prepared and connected to an electron accepting ZnPc by metal‐ligand, axial coordination of pyridyl in the 10 *meso*‐position of Cor to zinc metal center of Pc (Scheme 1).

## Results and Discussion

A widely extended method to obtain *trans*‐A_2_B Cor relies on the reaction of the desired dipyrromethane with a suitable aldehyde.^[14]^ Starting from mesitylbenzaldehyde for the synthesis of **py**‐**CorM_6_** and pentafluorobenzaldehyde for the synthesis of **py**‐**CorF_10_**, the corresponding dipyrromethanes were synthesized by a reaction with pyrrole, using the method already reported in the literature.^[15]^ Both 10‐*meso*‐pyridyl‐substituted Cor were obtained following an already reported procedure, aimed to the synthesis of *trans*‐A_2_B‐corroles bearing substituents with basic nitrogen atoms at the *meso*‐positions. Cor formation reaction involves the acid‐catalyzed condensation of a dipyrromethane with an aldehyde followed by oxidation with DDQ.^[16]^ Octakis(2‐ethylhexylsulfonyl) Zn(II)Pc (**ZnPcR_8_**) was prepared as already reported in the literature.^[11b]^

## NMR titrations

**Figure 1:**
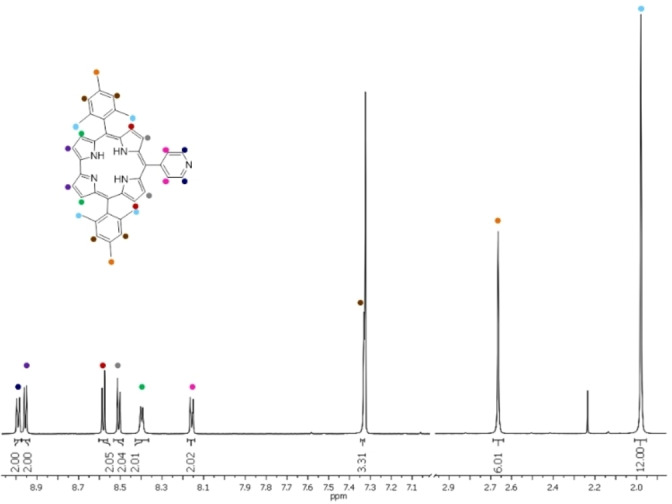
^1^H NMR spectrum of **py‐CorM_6_** in CDCl_3_ at room temperature. Full assignment obtained after combining COSY, ROESY, and ^1^H‐^13^C HSQC techniques.

**Figure 2:**
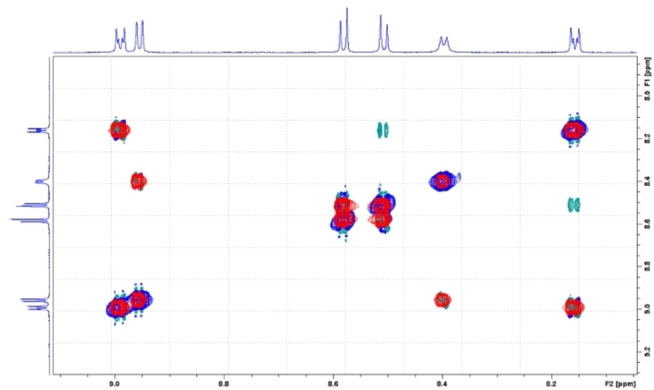
Combination of COSY (red) and ROESY (green and blue) analyses of **py‐CorM_6_** in CDCl_3_ at room temperature (focus on the *meso*‐pyridine and *β*‐pyrrolic signals). Green cross peaks indicate a dipolar correlation of protons at position 8 and12 with the two *ortho*‐pyridine protons.

**Figure 3:**
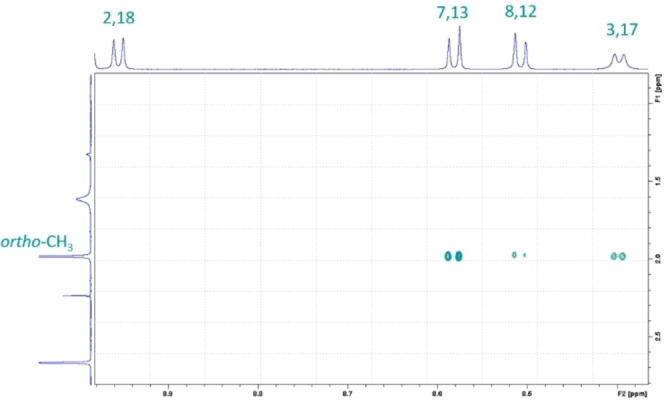
ROESY spectrum of the *β*‐pyrrolic positions of **py‐CorM_6_** in CDCl_3_ at room temperature. Protons at positions 2 and 18 lack any cross peak with the CH_3_ *ortho*‐functionalities of the *meso*‐phenyl groups.

Measurements were carried out in CDCl_3_ at 20 °C with a constant concentration of **py**‐**CorM_6_** (100 μM) and variable concentrations of **ZnPcR_8_**. To appreciate changes in the chemical shifts a complete assignment of **py**‐**CorM_6_** and **ZnPcR_8_** ⋅ **py**‐**CorM_6_** was necessary. The assigned ^1^H NMR spectrum of **py**‐**CorM_6_** is shown in Figure 1. Methyl resonances were assigned based on their different integration values in the aliphatic range from 1.98 and 2.66 ppm. Assignment of the protons in the *β*‐pyrrolic positions were done by combining the COSY and ROESY spectra. Protons at positions 2 and 18 were unambiguously found at 8.95 ppm. These protons are not subject to any dipolar interactions with CH_3_ *ortho*‐functionalities of the *meso*‐phenyl groups as they do not feature any cross peaks in the ROESY analyses (Figure 2). Any other assignment of, for example, the aromatic protons was possible by the combination of COSY and ROESY. Figure 3 shows the scalar couplings between the protons in the positions 2,18 and 3,17, and a cross‐peak due to a dipolar correlation of the 8,12 *β*‐pyrrolic protons with the two pyridine protons.

**Figure 4:**
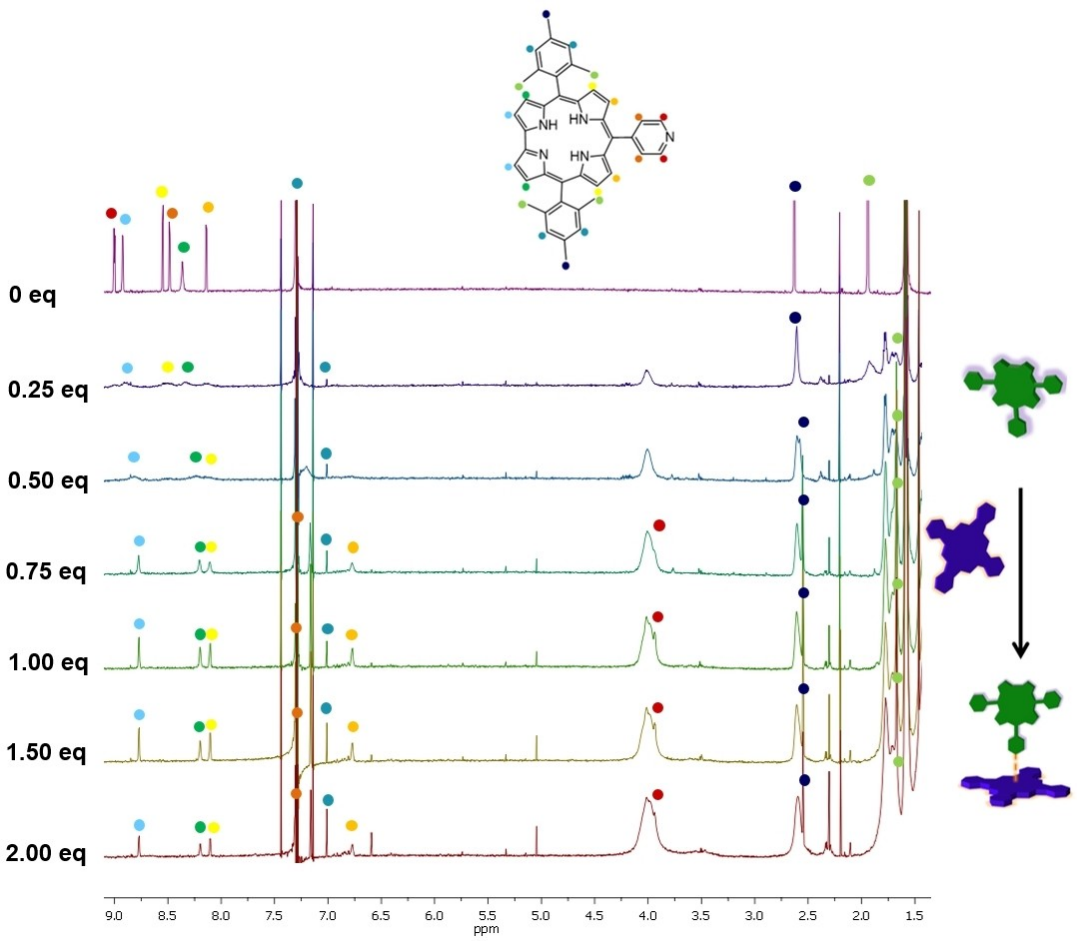
^1^H NMR titration assays of **py‐CorM_6_** with variable equivalents of **ZnPcR_8_** in CDCl_3_ at 20 °C.

**Figure 5:**
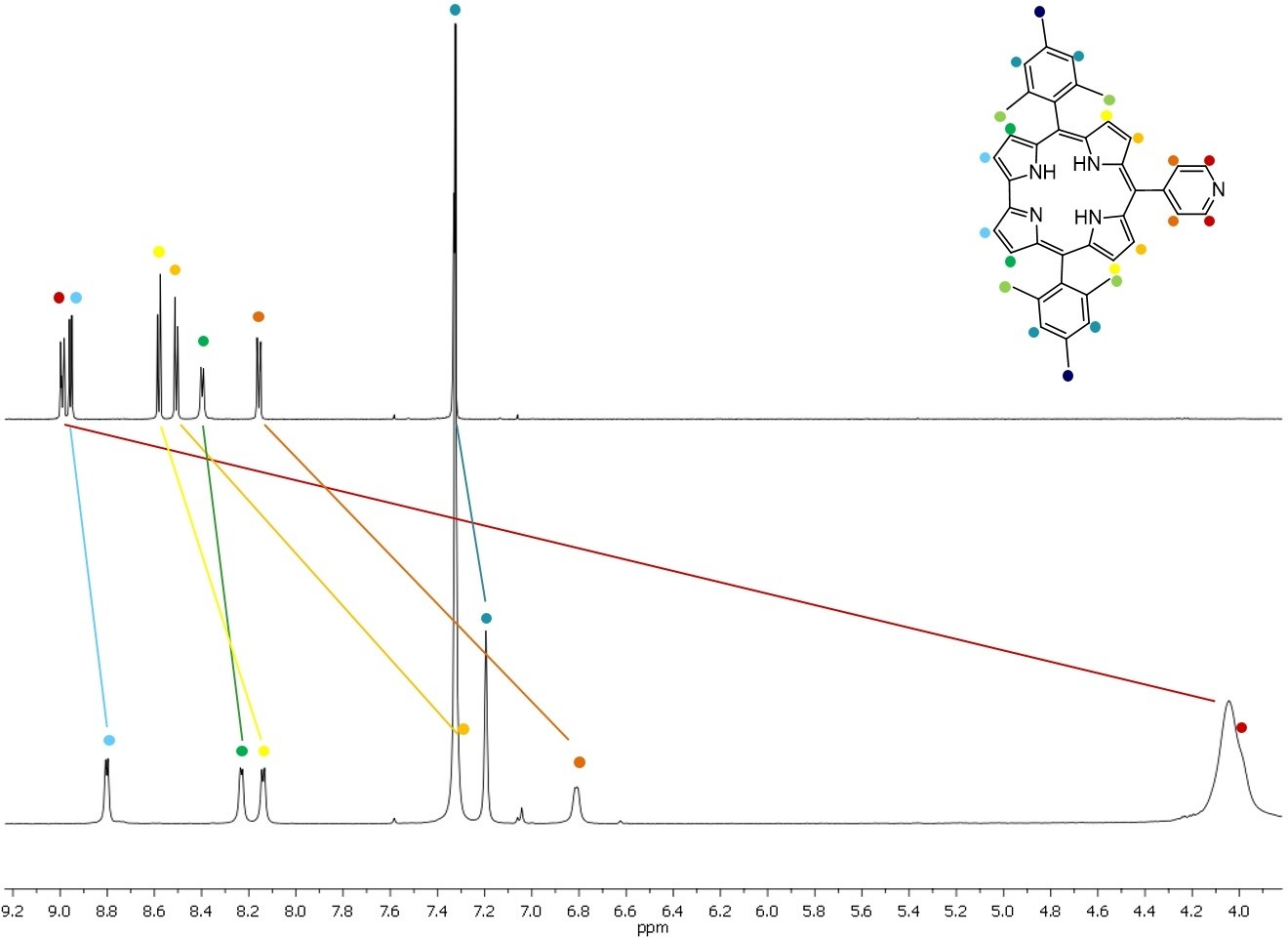
First (0 equiv. of **ZnPcR_8_**) and last (2 equiv. **ZnPcR_8_**) ^1^H NMR spectra of the titrations performed with **py‐CorM_6_** and **ZnPcR_8_**. Inset displays a table with the chemical shift perturbations of the **py‐CorM_6_** protons.

A typical titration of **py**‐**CorM_6_** with **ZnPcR_8_** is summarized in Figure 4. Upon addition of **ZnPcR_8_** a severe broadening of the peak line‐widths is observed, which renders the assignment of some protons rather difficult. Still, all peaks shift toward lower ppm with, however, different magnitudes. Changes become discernable at 0.25 equivalents of **ZnPcR_8_** and the pyridine protons, which are the closest to **ZnPcR_8_**, are those that are most affected by the aromatic ring current. To appreciate the Δ*δ* from Cor, 2‐dimensional analyses of **ZnPcR_8_** ⋅ **py**‐**CorM_6_** were performed using the same concentration for both of them, that is, 2.5 mm (Figures S4–7). The initial and the final step of the titration experiments are shown in Figure 5 next to the chemical shift perturbations experienced by **py**‐**CorM_6_**. Following the **ZnPcR_8_** ⋅ **py**‐**CorM_6_** formation, the chemical shift perturbation varies from about 0.08 all the way to 5.01 ppm.

From these experiments it is, however, evident that the concentrations were too high to determine the effective thermodynamic constant *K*_D_. In particular, the range of [Pc]/[Corr] was insufficient to cover the full curvature of the binding plot. As such, the signals seem to saturate already at 0.75 equivalents of Pc, which makes the value of the binding constant unreliable.

At, for example, 100 μm of **py**‐**CorM_6_**, the association constant is predicted to be greater or equal to 10^6^ m ^−1^. Overall, we feel that it represents a useful estimate for the exchange constant, *k*_exc_.

The different Δ*δ* values, which are tabled in the inset of Figure 5, stem from different exchange regimes that the Cor protons experience. Aliphatic mesityl protons (dark blue and light green dots in Figure 4) are barely affected by the electronic changes due to complexation. This results in a fast exchange, in which the peak intensities remain constant throughout the titrations. Protons belonging to the pyridine moiety and to the positions 7,13 and 8,12 of the corrole macrocycle (red, orange, light orange, and yellow dots in Figure 4) are the most affected by the interaction, and display a slow exchange behavior. Protons in the Cor 2,18, 3,17 positions, as well as the aromatic protons of the mesityl groups, which are marked in light blue, green, and blue in Figure 4, are affected by an intermediate exchange regime. Here, the resonance lines become very broad as the Pc is added and the system slowly equilibrates from the free macrocycle to the fully coordinated Pc‐Corr. As such, we gather information about how to estimate *k*_exc_ from the aromatic protons belonging to the mesityl group: prior to any **ZnPcR_8_** addition, their signal was covered by the solvent (Figure 5). Throughout the titration experiments, a shift toward lower ppm was evident and the intensities of the new peaks directly relate to the concentration of **ZnPcR_8_** ⋅ **py**‐**CorM_6_**. In the case of intermediate exchange conditions, k_exc_ is approximated by takings the differences of chemical shifts between the free and bound corrole. The chemical shift perturbation was found to be 0.148 ppm, resulting in an exchange rate value of *k*_exc_ ≃ 103 s^−1^.

To assist in the interpretation of the NMR data and to gather more quantitative information about the binding phenomenon, different sets of physicochemical experiments were performed. To elucidate the electronic behaviour of Cor and its function as light harvester and electron donor to power energy and/or electron transfer upon photoexcitation, we complemented our investigation by probing **py**‐**CorF_10_**.

## Electrochemical characterization

Square‐wave and cyclic voltammograms of **py**‐**CorM_6_** and **py**‐**CorF_10_** next to **ZnPcR_8_** were recorded in dichloromethane with 0.1 m [Bu_4_N][PF_6_] as electrolyte (Figures S8‐10). **py**‐**CorM_6_** and **py**‐**CorF_10_** both feature single reductions and oxidations, which are *quasi* reversible and reversible, respectively, at −1.85 and +0.34 V versus Fc/Fc^+^ for **py**‐**CorM_6_** as well as at −1.58 and +0.58 V versus Fc/Fc^+^ for **py**‐**CorF_10_**. Importantly, **py**‐**CorF_10_** is by far a better electron acceptor and a poorer electron donor than **py**‐**CorM_6_**, due to the electron‐withdrawing substituents. In contrast, four reductions and a single oxidation are seen for **ZnPcR_8_**, that is, at −0.74, −0.99, −1.09, and −1.30 V versus Fc/Fc^+^ as well as at +0.90 V versus Fc/Fc^+^. All **ZnPcR_8_**‐based values, with the exception of the second reduction, are in agreement with those determined for a ZnPc‐reference.^[10b]^ Reductions and oxidations are summarised in Table 1. Combining **py**‐**CorM_6_** and **py**‐**CorF_10_** as electron donors and **ZnPcR_8_** as electron acceptor, respectively, and realizing **ZnPcR_8_** ⋅ **py**‐**CorM_6_** and **ZnPcR_8_** ⋅ **py**‐**CorF_10_** results into charge‐separated state energies of 1.08 and 1.32 eV, respectively.

**Table 1:** Reductions and oxidations of **py‐CorM_6_**, **py‐CorF_10_**, and **ZnPcR_8_** in V vs. Fc/Fc^+^ with 0.1 m [Bu_4_N][PF_6_] as electrolyte.

	4th red.	3rd red.	2nd red.	1st red.	1st ox.
**py‐CorM_6_**				−1.85	+0.34
**py‐CorF_10_**				−1.58	+0.58
**ZnPcR_8_**	−1.30	−1.09	−0.99	−0.74	+0.90

**Figure 6:**
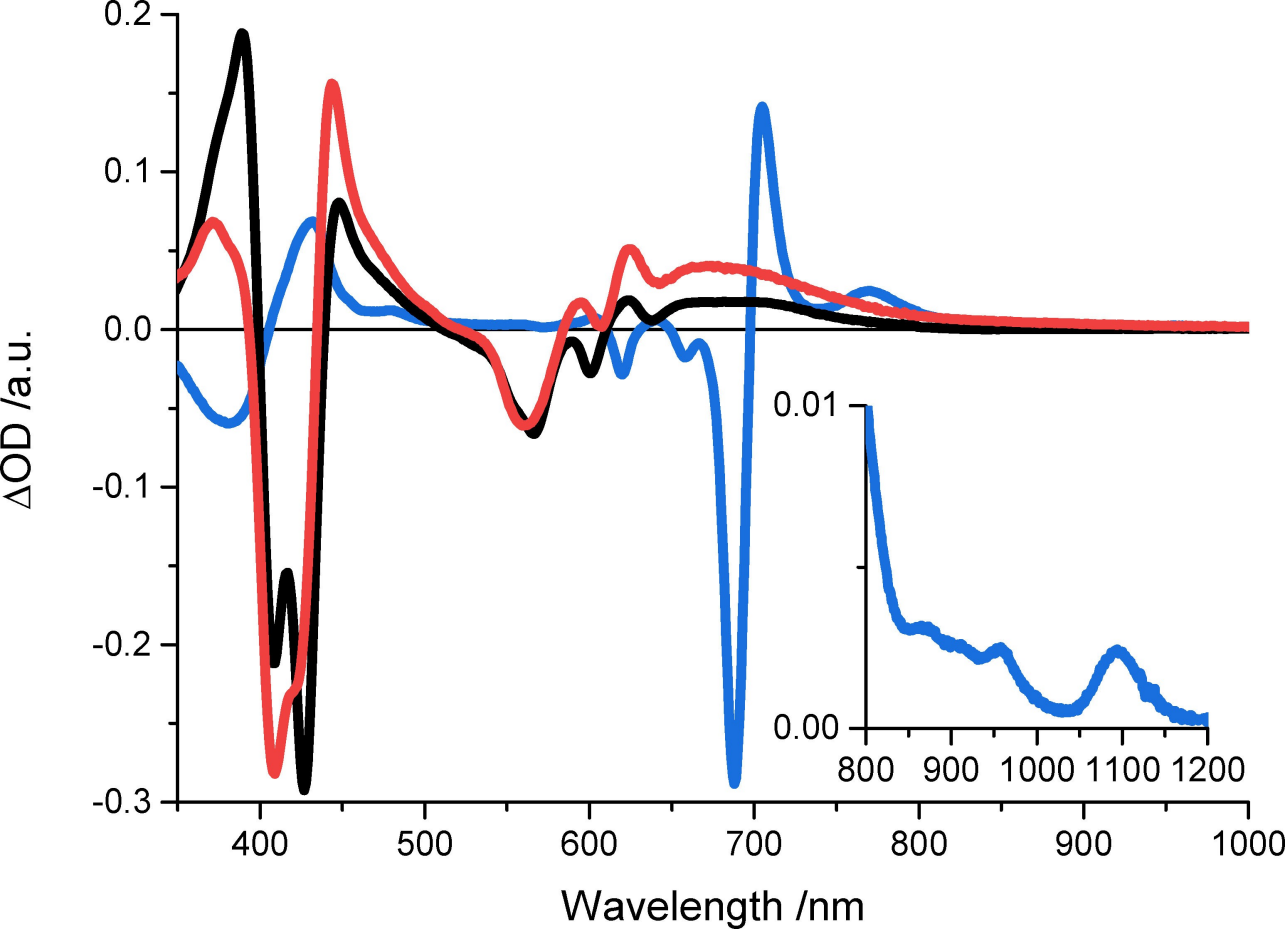
Differential absorption spectra obtained upon one‐electron oxidation of **py‐CorM_6_** (black) and **py‐CorF_10_** (red) at a bias of +0.9 V vs. Ag‐wire in de‐aerated dichloromethane with 0.1 m [Bu_4_N][PF_6_] as electrolyte and upon one‐electron reduction of **ZnPcR_8_** (blue) at a bias of −0.8 V vs. Ag‐wire in de‐aerated o‐dichlorobenzene with 0.1 m [Bu_4_N][ClO_4_] as electrolyte.

Spectro‐electrochemical experiments were conducted for **py**‐**CorM_6_** and **py**‐**CorF_10_** in dichloromethane, while *o*‐dichlorobenzene turned out to be better suited for **ZnPcR_8_** (Figure 6). **py**‐**CorM_6_** and **py**‐**CorF_10_** feature similar differential spectra upon their one‐electron oxidations with 370–390, 445, 625, and 670 nm maxima, as well as 410, 425, 565, and 600–605 nm minima. **ZnPcR_8_** reveals upon its one‐electron reduction maxima at 430, 600, 640, 705, and 770 nm, while minima are seen at 380, 620, 660, and 690 nm. In the near infrared, the maxima are at 960 and 1095 nm.

## Photophysical characterization

**Figure 7:**
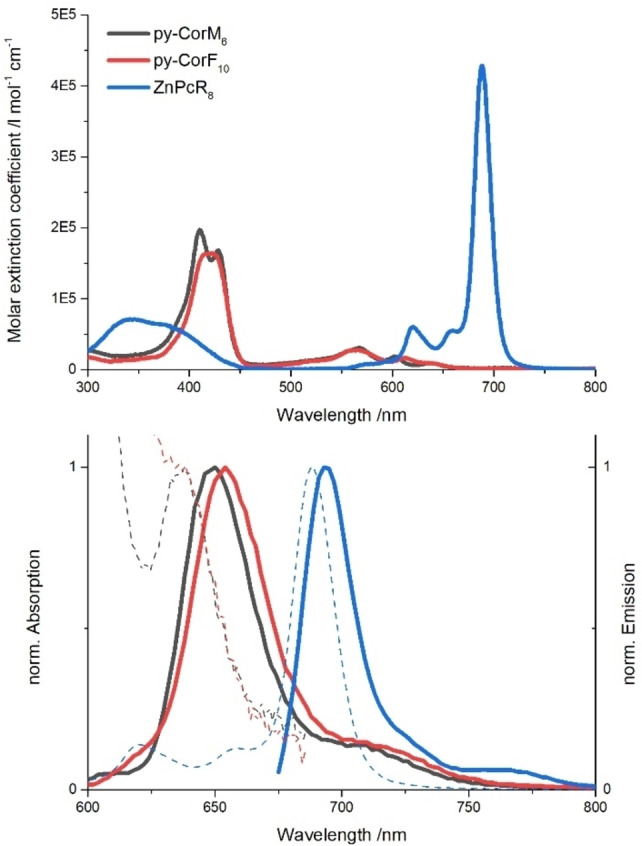
Absorption spectra at the top and normalized fluorescence spectra at the bottom of **py‐CorM_6_** (black), **py‐CorF_10_** (red), and **ZnPcR_8_** (blue) in toluene at room‐temperature including the corresponding absorption spectra (dashed lines).

Steady‐state absorption and fluorescence spectra of **py**‐**CorM_6_**, **py**‐**CorF_10_**, and **ZnPcR_8_** were recorded in toluene (Figure 7). Hereby, the absorption spectra of **py**‐**CorM_6_** and **py**‐**CorF_10_** are dominated by two strong Soret‐band absorptions at 410/429 and 415/424 nm, respectively.

In each case, four weak Q‐band absorptions evolve in the range from 510 to 640 nm. **ZnPcR_8_** features weak Soret‐band absorptions in the 320 to 400 nm range and a multitude of strong Q‐band absorptions, which peak at 688 nm. The latter complement those of **py**‐**CorM_6_** and **py**‐**CorF_10_**. When turning to the fluorescence spectra, the respective maxima are 650 nm (**py**‐**CorM_6_**), 654 nm (**py**‐**CorF_10_**), and 694 nm (**ZnPcR_8_**). They exhibit nearly the same energies and quantum yields. In particular, the quantum yields are 0.29, 0.19, and 0.29 for **py**‐**CorM_6_**, **py**‐**CorF_10_**, and **ZnPcR_8_**, respectively. All relevant values are summarised in Table 2.

**Table 2:** Absorption maxima, molar extinction coefficients *ϵ*_max_, fluorescence maxima, and fluorescence quantum yields *Φ*_Fl_ in toluene. Tetraphenylporphyrin (H_2_TPP) and zinc tetra‐*tert*‐butyl‐phthalocyanine (ZnT*t*BuPc) were used as the respective references for Cor and ZnPc.^[17]^

	Absorption_max_	*ϵ*_max_/cm^−1^ m ^−1^	Fluorescence_max_	*Φ*_Fl_
**py‐CorM_6_**	410 nm	1.97×10^5^	650 nm	0.29
**py‐CorF_10_**	424 nm	1.64×10^5^	654 nm	0.19
**ZnPcR_8_**	688 nm	4.28×10^5^	694 nm	0.29

**Figure 8:**
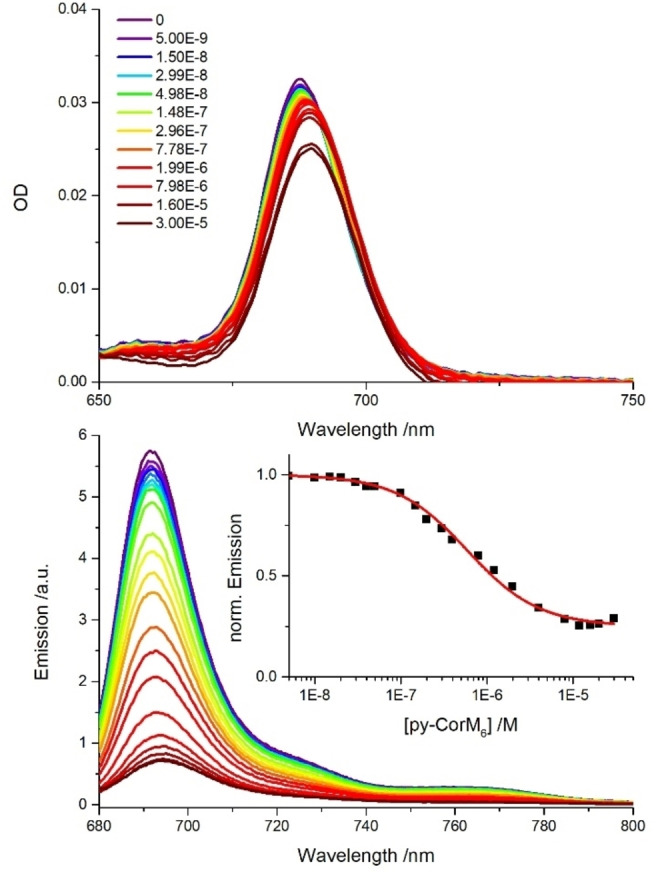
Absorption spectra at the top and fluorescence spectra at the bottom of **ZnPcR_8_** (1×10^−7^ m) upon addition of variable **py‐CorM_6_** concentrations (0‐3×10^−5^ m) in toluene at room‐temperature. Inset displays the normalized **ZnPcR_8_** fluorescence to determine the binding constant.

Formation of **ZnPcR_8_** ⋅ **py**‐**CorM_6_** and **ZnPcR_8_** ⋅ **py**‐**CorF_10_** was followed and analyzed in steady‐state absorption and fluorescence assays. Variable concentrations of either **py**‐**CorM_6_** or **py**‐**CorF_10_** were added to a solution of **ZnPcR_8_**, whose concentration was kept constant. Throughout these titrations the **ZnPcR_8_**‐centered Q‐band absorption at 688 nm red‐shifts and attenuates. Hand‐in‐hand with the red‐shifted absorption is a **ZnPcR_8_**‐centered fluorescence that is subject to a non‐linear quenching. In this regard, the association constants are 1.8±0.2×10^6^ m ^−1^ for **ZnPcR_8_** ⋅ **py**‐**CorM_6_** as well as 6.0±1.1×10^5^ m ^−1^ for **ZnPcR_8_** ⋅ **py**‐**CorF_10_** (Figures 8 & S11). Employing chlorobenzene led like toluene to appreciable changes with comparable constants on the order of 10^6^ m ^−1^ (Figures S14 & S15).

Measurements with a Cor‐reference resulted in a similar non‐linear fluorescence quenching. Even the association constant is in the same range, that is, 2.8±0.7×10^6^ m ^−1^ and is, thus, consistent with the NMR data (Figure S16). When using a ZnPc‐reference, which features *tert*‐butyl groups, the fluorescence quenching was much weaker (Figures S18 & S19). The association constants are 2.7±1.1×10^5^ and 1.4±0.3×10^5^ m ^−1^ for ZnPc ⋅ **py**‐**CorM_6_** and ZnPc ⋅ **py**‐**CorF_10_**, respectively. Titrations in anisole and ethanol led to significantly weaker binding with association constants in the range of 10^4^ m ^−1^ rather than 10^5^ or 10^6^ m ^−1^ (Figures S12, S13 & S17).

Finally, temperature‐dependent titration experiments were performed with mixtures of **py**‐**CorM_6_**, **py**‐**CorF_10_**, and Cor‐reference as well as **ZnPcR_8_** in toluene in the range from 20 to 60 °C (Figures S20–22). Overall, the association constants decreased as the temperatures were increased. This is well in line with the notion that the formation of **ZnPcR_8_** ⋅ **py**‐**CorM_6_** and **ZnPcR_8_** ⋅ **py**‐**CorF_10_** is exergonic. Increasing the temperature is also associated with a slightly weaker fluorescence quenching. This prompts to a slower, but still exergonic separation of charges, while the intrinsic **ZnPcR_8_** fluorescence remains unaffected.

Insights into the excited state dynamics were gathered by photo‐exciting **py**‐**CorM_6_**/**py**‐**CorF_10_** and **ZnPcR_8_** at 430 and 676 nm, respectively. In the case of **py**‐**CorM_6_**, the first three transient species feature characteristics of the singlet excited state. These were maxima at 460 and 800 nm next to minima at 568 and 604 nm (Figure S23). The first two relaxations take place with 3 and 47 ps and transform the initially vibrationally and electronically hot singlet excited state into the fully relaxed and fluorescent singlet excited state. The latter transforms ultimately into the triplet excited state with its red‐shifted 455 nm maximum. The singlet excited state of **py**‐**CorF_10_** features a minimum at 565 nm and maxima at 460, 750 and <1250 nm. Initially, it is the vibrationally and electronically hot singlet excited state that is formed. It relaxes with 24 ps before it decays into the triplet excited state, for which the latter two maxima are missing, within several ns (Figure S24). For **ZnPcR_8_**, a vibrationally and electronically hot singlet excited state relaxes with 14 and 555 ps to afford a relaxed singlet excited state. Signatures of the latter are maxima at 510 and 887 nm as well as a ground state bleaching at 695 nm. The corresponding triplet excited state with maxima at 440 and 795 nm and ground state bleaching at 692 nm (Figure S25) is formed with 2.3 ns before it decays to the ground state.

**Figure 9:**
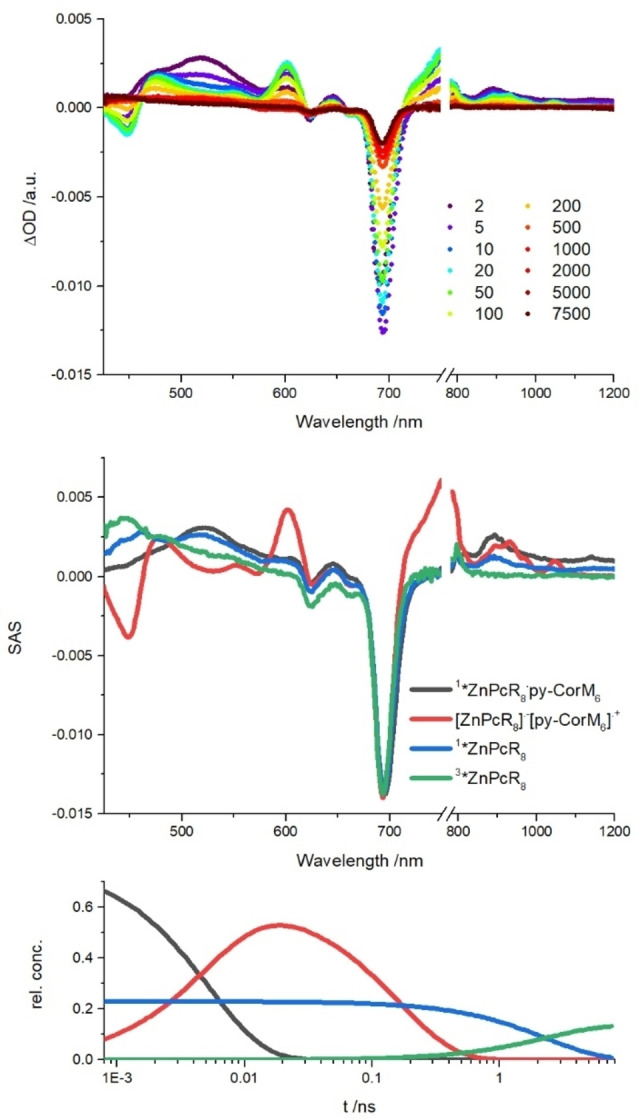
Differential absorption spectra on the top obtained upon femtosecond flash‐photolysis (676 nm) of a 10 : 1 mixture of **py‐CorM_6_** and **ZnPcR_8_** in de‐aerated toluene at room‐temperature with time delays between 2 and 7500 ps. Species associated spectra of ^**1**^ *ZnPcR_8_ ⋅ **py‐CorM_6_** (black), **[ZnPcR_8_]** ^.**−**^ ⋅ **[py‐CorM_6_]** ^.**+**^ (red), ^**1**^ *ZnPcR_8_ (blue), and ^3^*ZnPcR_8_ (green) in the center and relative concentration profiles of the transient species at the bottom.

Mixtures of **py**‐**CorM_6_** and **ZnPcR_8_** as well as **py**‐**CorF_10_** and **ZnPcR_8_** were probed by means of 430 and 676/670 nm photo‐excitation experiments, respectively. Using an excess of **ZnPcR_8_**, implies the 676/670 nm co‐excitation of either **ZnPcR_8_** ⋅ **py**‐**CorM_6_** or **ZnPcR_8_** **⋅ py**‐**CorF_10_**, on one hand, and **ZnPcR_8_**, on the other hand. In such a case, a model with a total of four species was fully sufficient to fit the raw data. Two of them relate to **ZnPcR_8_** ⋅ **py**‐**CorM_6_**/**ZnPcR_8_** ⋅ **py**‐**CorF_10_** and the other two to **ZnPcR_8_**. As a leading example, 676 nm photo‐excitation of **ZnPcR_8_** ⋅ **py**‐**CorM_6_** leads in toluene to the **ZnPcR_8_**‐centered singlet excited state formation (Figure 9). It decays with 5 ps and yields in 77 % a charge separated state, that is, [**ZnPcR_8_**]^.−^ ⋅ [**py**‐**CorM_6_**]^.+^. Evidence for the charge separation comes from the observation of the one‐electron reduced form of **ZnPcR_8_** with maxima at 600, 750–780, 935, 970, and 1050 nm and of the electron oxidized form of **py**‐**CorM_6_** with a ground state bleaching and a maximum at 450 and 480 nm, respectively. It reinstates the ground state with 164 ps. Meanwhile non‐complexed **ZnPcR_8_** undergoes intersystem crossing, by which the singlet excited state interconverts into the triplet excited state with 2.3 ns. Similar results are obtained for **ZnPcR_8_** ⋅ **py**‐**CorF_10_** with lifetimes of 12 and 1400 ps for the singlet excited state and the [**ZnPcR_8_**]^.−^ ⋅ [**py**‐**CorF_10_**]^.+^charge separated state, respectively (Figure S26). Corresponding experiments in anisole are virtually identical. Upon photo‐excitation at 670 nm, 6 and 11 ps are the **ZnPcR_8_**‐centered singlet excited state lifetimes in **ZnPcR_8_** ⋅ **py**‐**CorM_6_** and **ZnPcR_8_** ⋅ **py**‐**CorF_10_**, respectively. The corresponding charge‐separated states are formed in 84 % for [**ZnPcR_8_**]^.−^ ⋅ [**py**‐**CorM_6_**]^.+^ and 94 % for [**ZnPcR_8_**]^.−^ ⋅ [**py**‐**CorF_10_**]^.+^. The decays are 29 and 127 ps as the ground‐states are recovered (Figures S29‐30). All relevant lifetimes are put together in Table 3. Charge separation was also successful with 75 % yield upon photoexcitation at 430 nm in the presence of an excess of **ZnPcR_8_**. In these cases, the starting point was, however, mostly **py**‐**CorM_6_**‐/**py**‐**CorF_10_**‐centered (Figures S27–28).

**Table 3:** Charge separation and recombination for **ZnPcR_8_** ⋅ **py‐CorM_6_** and **ZnPcR_8_** ⋅ **py‐CorF_10_** in toluene and anisole.

	ZnPcR_8_ ⋅ py‐CorM_6_		ZnPcR_8_ ⋅ py‐CorF_10_	
toluene	5 ps	164 ps	12 ps	1400 ps
anisole	6 ps	29 ps	11 ps	127 ps

## Conclusion

We have explored supramolecular interactions between two different corroles (Cor) bearing either electron‐donating or electron‐withdrawing groups, with an electron‐withdrawing‐substituted phthalocyanine (Pc). Strong chemical‐shift perturbations were observed in ^1^H NMR analyses of titrations, up to 5 ppm. Qualitative information regarding the binding constant and exchange constant was gathered. To assist the interpretation of the NMR data, excited‐state properties were studied. Photoexcitation of either the Pc or Cor is the starting point to a fast charge‐separation in combination with a slow charge‐recombination. The latter reaches well into the thousands of picoseconds. Importantly, even if perfluorinated, Cor proves to be an excellent electron donor, highlighting its utility as sensitizer in light‐harvesting applications.

## Experimental Section

**Material and general methods**: Unless otherwise stated, all reagents and solvents were obtained from commercial suppliers and used without further purification. Chromatography: Thin layer chromatography (TLC) analyses were performed on aluminum sheets coated with silica gel 60 F254 or neutral alumina 60 F254 (Merck). TLC analyses were carried out with an UV lamp of 254 and 365 nm. Column chromatography was carried out using silica gel Merck‐60 (230–400 mesh, 60 a), Sigma‐Aldrich (70–230 mesh, 60 a) and and neutral alumina (Merk, Brockmann Grade III) as the solid support. Eluents and relative proportions are indicated for each particular case. Nuclear magnetic resonance (NMR): for structural assignments 1H and 13 C spectra were recorded on a Bruker Avance spectrometer operating at 700 MHz for 1H, equipped with a 5 mm inverse TXI probe and z‐axis gradients. Chemical shifts are reported on the *δ* scale (ppm), where the residual solvent signal has been used as an internal reference. The NMR spectra were processed using the software MestreNova 11.0.

Electrochemistry: Square‐wave and cyclic voltammetry were performed on a three‐electrode setup (WE: C, CE: Pt, RE: Ag) with Fc/Fc^+^ as an internal standard. Solvents were de‐aerated using Ar. Spectroelectrochemistry: A three electrode‐setup (WE: Pt‐mesh, CE: Pt, RE: Ag) was used together with a custom‐made glass cell. Absorption was measured through the transparent WE with an Agilent CARY 5000 UV‐Vis‐NIR spectrophotometer. Solvents were de‐aerated using Ar. Steady‐state Absorption & Fluorescence: 10x10 mm quartz cuvettes were used for both methods. Absorption was measured at a Perkin Elmer Lambda 2 spectrometer, a Horiba Jobin Yvon Fluoromax 3 was used for Fluorescence experiments.

**Transient absorption**: Spectra were obtained with a Ti:sapphire laser system CPA‐2101 (Clark‐MXR, Inc.) paired with an Ultrafast Inc. Helios TAPPS‐transient absorption pump probe spectroscopy detection unit (pump: 1 kHz repetition, 150 fs pulse width). 2×10 mm quartz cells were used while spectra were acquired with an Ultrafast Systems HELIOS transient absorption spectrometer.

**Synthetic procedures**: **Py‐CorF_10_**, **py‐CorM_6_** [^15^, ^16^] and **ZnPcR_8_** ^[11b]^ were prepared following literature procedures.

## Conflict of interest

The authors declare no conflict of interest.

## Supporting information

As a service to our authors and readers, this journal provides supporting information supplied by the authors. Such materials are peer reviewed and may be re‐organized for online delivery, but are not copy‐edited or typeset. Technical support issues arising from supporting information (other than missing files) should be addressed to the authors.

Supporting Information

## References

[1a] B. Alberts, B. Alberts, A. Johnson, J. Lewis, M. C. Raff, K. Roberts, P. Walter, J. H. Wilson, T. Hunt, *Molecular Biology of the Cell*, Garland Science, New York, **2008**;

[1b] D. K. Dogutan , D. G. Nocera , Acc. Chem. Res. 2019, 52, 3143. 31593438 10.1021/acs.accounts.9b00380

[2] K. Kato , T. Shinoda , R. Nagao , S. Akimoto , T. Suzuki , N. Dohmae , M. Chen , S. I. Allakhverdiev , J. R. Shen , F. Akita , N. Miyazaki , T. Tomo , Nat. Commun. 2020, 11, 238. 31932639 10.1038/s41467-019-13898-5 PMC6957486

[3a] V. Balzani , A. Credi , M. Venturi , ChemSusChem 2008, 1, 26; 18605661 10.1002/cssc.200700087

[3b] R. E. Blankenship , Molecular Mechanisms of Photosynthesis, 2nd Edition, John Wiley & Sons, 2014;

[3c] B. R. Green , W. W. Parson , Light-harvesting antennas in photosynthesis, Springer, Dordrecht, London 2011;

[3d] N. Nelson , C. F. Yocum , Annu. Rev. Plant Biol. 2006, 57, 521; 16669773 10.1146/annurev.arplant.57.032905.105350

[3e] J. H. Wilson, T. Hunt, *Molecular biology of the cell, 4th edition : a problems approach*, Garland Science, New York, London **2002**.

[4a] M. Hao , G. Sun , M. Zuo , Z. Xu , Y. Chen , X.-Y. Hu , L. Wang , Angew. Chem. Int. Ed. 2020, 59, 10095; 10.1002/anie.201912654 31625651

[4b] G. Sun , M. Zuo , W. Qian , J. Jiao , X.-Y. Hu , L. Wang , Green Synth. Catal. 2021, 2, 32;

[4c] Z. Zhang , Z. Zhao , Y. Hou , H. Wang , X. Li , G. He , M. Zhang , Angew. Chem. Int. Ed. 2019, 58, 8862; 10.1002/anie.201904407 PMC6854906 31034686

[4d] Y. Tao , C. Yang , J. Qin , Chem. Soc. Rev. 2011, 40, 2943; 21369622 10.1039/c0cs00160k

[4e] S.-C. Lo , P. L. Burn , Chem. Rev. 2007, 107, 1097; 17385927 10.1021/cr050136l

[4f] S. Saha , J. F. Stoddart , Chem. Soc. Rev. 2007, 36, 77; 17173147 10.1039/b607187b

[4g] H. Q. Peng , L. Y. Niu , Y. Z. Chen , L. Z. Wu , C. H. Tung , Q. Z. Yang , Chem. Rev. 2015, 115, 7502; 26040205 10.1021/cr5007057

[4h] J. M. Yuen , J. R. Diers , E. J. Alexy , A. Roy , A. K. Mandal , H. S. Kang , D. M. Niedzwiedzki , C. Kirmaier , J. S. Lindsey , D. F. Bocian , D. Holten , J. Phys. Chem. A 2018, 122, 7181; 30152691 10.1021/acs.jpca.8b06815

[4i] A. Agresti , B. Berionni Berna , S. Pescetelli , A. Catini , F. Menchini , C. Di Natale , R. Paolesse , A. Di Carlo , Adv. Funct. Mater. 2020, 30, 2003790;

[4j] D. Guzmán , I. Papadopoulos , G. Lavarda , P. R. Rami , R. R. Tykwinski , M. S. Rodríguez-Morgade , D. M. Guldi , T. Torres , Angew. Chem. Int. Ed. 2021, 60, 1474; 10.1002/anie.202011197 PMC7839765 33002284

[5] D. Gust , T. A. Moore , A. L. Moore , Acc. Chem. Res. 1993, 26, 198.

[6a] D. Wróbel , Mol. Cryst. Liq. Cryst. 2016, 627, 4;

[6b] G. Bottari , G. de la Torre , D. M. Guldi , T. Torres , Coord. Chem. Rev. 2021, 428, 213605;

[6c] Z.-W. Li , J.-J. Yang , X.-Y. Liu , W.-H. Fang , H. Wang , G. Cui , Chem. Eur. J. 2021, 27, 4159; 33372312 10.1002/chem.202004850

[6d] E. Anaya-Plaza , J. Joseph , S. Bauroth , M. Wagner , C. Dolle , M. Sekita , F. Gröhn , E. Spiecker , T. Clark , A. de la Escosura , D. M. Guldi , T. Torres , Angew. Chem. Int. Ed. 2020, 59, 18786; 10.1002/anie.202006014 PMC7590087 32652750

[6e] M. Urbani , M.-E. Ragoussi , M. K. Nazeeruddin , T. Torres , Coord. Chem. Rev. 2019, 381, 1.

[7a] A. Ghosh , Chem. Rev. 2017, 117, 3798; 28191934 10.1021/acs.chemrev.6b00590

[7b] G. de la Torre , C. G. Claessens , T. Torres , Chem. Commun. 2007, 2000; 10.1039/b614234f 17713062

[7c] D. Wöhrle , Adv. Mater. 1993, 5, 942.

[8a] R. Paolesse , A. Marini , S. Nardis , A. Froiio , F. Mandoj , D. J. Nurco , L. Prodi , M. Montalti , K. M. Smith , J. Porphyrins Phthalocyanines 2003, 07, 25;

[8b] B. Ventura , A. Degli Esposti , B. Koszarna , D. T. Gryko , L. Flamigni , New J. Chem. 2005, 29, 1559;

[8c] T. Ding , E. A. Alemán , D. A. Modarelli , C. J. Ziegler , J. Phys. Chem. A 2005, 109, 7411. 16834109 10.1021/jp052047i

[9a] N. Kobayashi , H. Ogata , N. Nonaka , E. A. Luk′yanets , Chem. Eur. J. 2003, 9, 5123; 14562330 10.1002/chem.200304834

[9b] D. Chahraoui , P. Valat , J. Kossanyi , Res. Chem. Intermed. 1992, 17, 219.

[10a] Y. Fang , Z. Ou , K. M. Kadish , Chem. Rev. 2017, 117, 3377; 28009499 10.1021/acs.chemrev.6b00546

[10b] L. Flamigni , D. T. Gryko , Chem. Soc. Rev. 2009, 38, 1635. 19587958 10.1039/b805230c

[11a] A. R. M. Soares , M. V. Martínez-Díaz , A. Bruckner , A. M. V. M. Pereira , J. P. C. Tomé , C. M. A. Alonso , M. A. F. Faustino , M. G. P. M. S. Neves , A. C. Tomé , A. M. S. Silva , J. A. S. Cavaleiro , T. Torres , D. M. Guldi , Org. Lett. 2007, 9, 1557; 17378573 10.1021/ol0703635

[11b] O. Trukhina , M. Rudolf , G. Bottari , T. Akasaka , L. Echegoyen , T. Torres , D. M. Guldi , J. Am. Chem. Soc. 2015, 137, 12914; 26401549 10.1021/jacs.5b06454

[11c] A. Hausmann , A. R. M. Soares , M. V. Martínez-Díaz , M. G. P. M. S. Neves , A. C. Tomé , J. A. S. Cavaleiro , T. Torres , D. M. Guldi , Photochem. Photobiol. Sci. 2010, 9, 1027; 20508890 10.1039/c0pp00060d

[11d] L. M. O. Lourenço , A. Hausmann , C. Schubert , M. G. P. M. S. Neves , J. A. S. Cavaleiro , T. Torres , D. M. Guldi , J. P. C. Tomé , ChemPlusChem 2015, 80, 832. 31973330 10.1002/cplu.201500005

[12] B. B. Berna , B. Platzer , M. Wolf , G. Lavarda , S. Nardis , P. Galloni , T. Torres , D. M. Guldi , R. Paolesse , Chem. Eur. J. 2020, 26, 13451. 32293078 10.1002/chem.202001442 PMC7693288

[13a] M. S. Rodríguez-Morgade , M. E. Plonska-Brzezinska , A. J. Athans , E. Carbonell , G. de Miguel , D. M. Guldi , L. Echegoyen , T. Torres , J. Am. Chem. Soc. 2009, 131, 10484; 19722625 10.1021/ja902471w

[13b] M. Krug , C. Stangel , A. Zieleniewska , T. Clark , T. Torres , A. G. Coutsolelos , D. M. Guldi , ChemPhysChem 2019, 20, 2806; 31471925 10.1002/cphc.201900780

[13c] G. Rotas , M. B. Thomas , R. Canton-Vitoria , F. D′Souza , N. Tagmatarchis , Chem. Eur. J. 2020, 26, 6652; 32159249 10.1002/chem.202000174

[13d] D. Badgurjar , S. Seetharaman , F. D′Souza , R. Chitta , Chem. Eur. J. 2021, 27, 2184; 33107661 10.1002/chem.202004262

[13e] N. Zarrabi , C. O. Obondi , G. N. Lim , S. Seetharaman , B. G. Boe , F. D′Souza , P. K. Poddutoori , Nanoscale 2018,10, 20723. 30398274 10.1039/c8nr06649c

[14] R. Orłowski , D. Gryko , D. T. Gryko , Chem. Rev. 2017, 117, 3102. 27813401 10.1021/acs.chemrev.6b00434

[15] J. K. Laha , S. Dhanalekshmi , M. Taniguchi , A. Ambroise , J. S. Lindsey , Org. Process Res. Dev. 2003, 7, 799.

[16] D. T. Gryko , K. E. Piechota , J. Porphyrins Phthalocyanines 2002, 06, 81.

[17] P. G. Seybold , M. Gouterman , J. Mol. Spectrosc. 1969, 31, 1.

