# Supplementary material for: Exploring the Association of Electron‐Donating Corroles with Phthalocyanines as Electron Acceptors
Source: Chemistry. 2022 Feb 10;28(13):e202103891. doi: 10.1002/chem.202103891 (PMC9306480; doi:10.1002/chem.202103891)
Supplement: Supplementary file 1 — Supporting Information [file CHEM-28-0-s001.pdf]

Chemistry–A European Journal

Supporting Information

Exploring the Association of Electron-Donating Corroles with Phthalocyanines as Electron Acceptors

Benedikt Platzer, Beatrice Berionni Berna, Martina Bischetti, Daniel O. Cicero, Roberto Paolesse, Sara Nardis,* Tomás Torres,* and Dirk M. Guldi*

Table of Contents

1. NMR spectra..S1

2. Cyclic and pulse voltammetry..S8

3. Photophysical measurements...S11

1. NMR SPECTRA

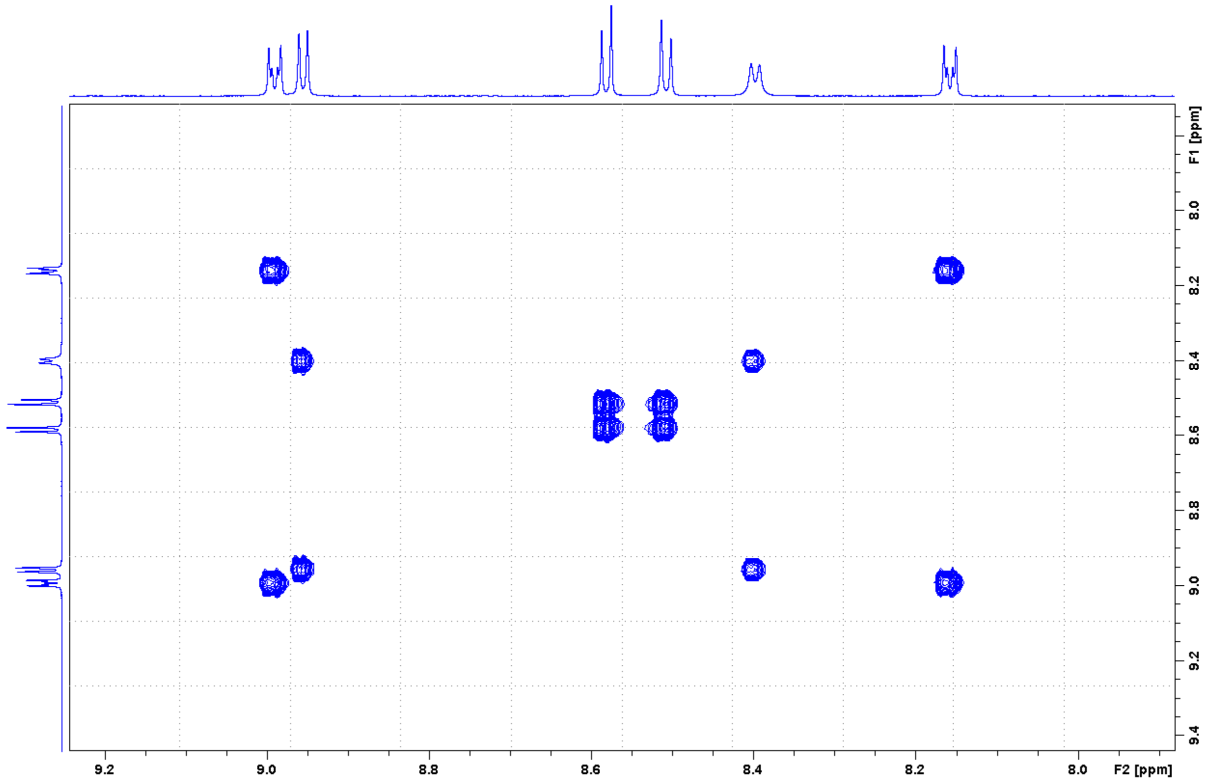

Figure S1. COSY spectrum of **py-CorM$_6$** in $CDCl_3$ at room temperature (focus on the β-pyrrolic signals).

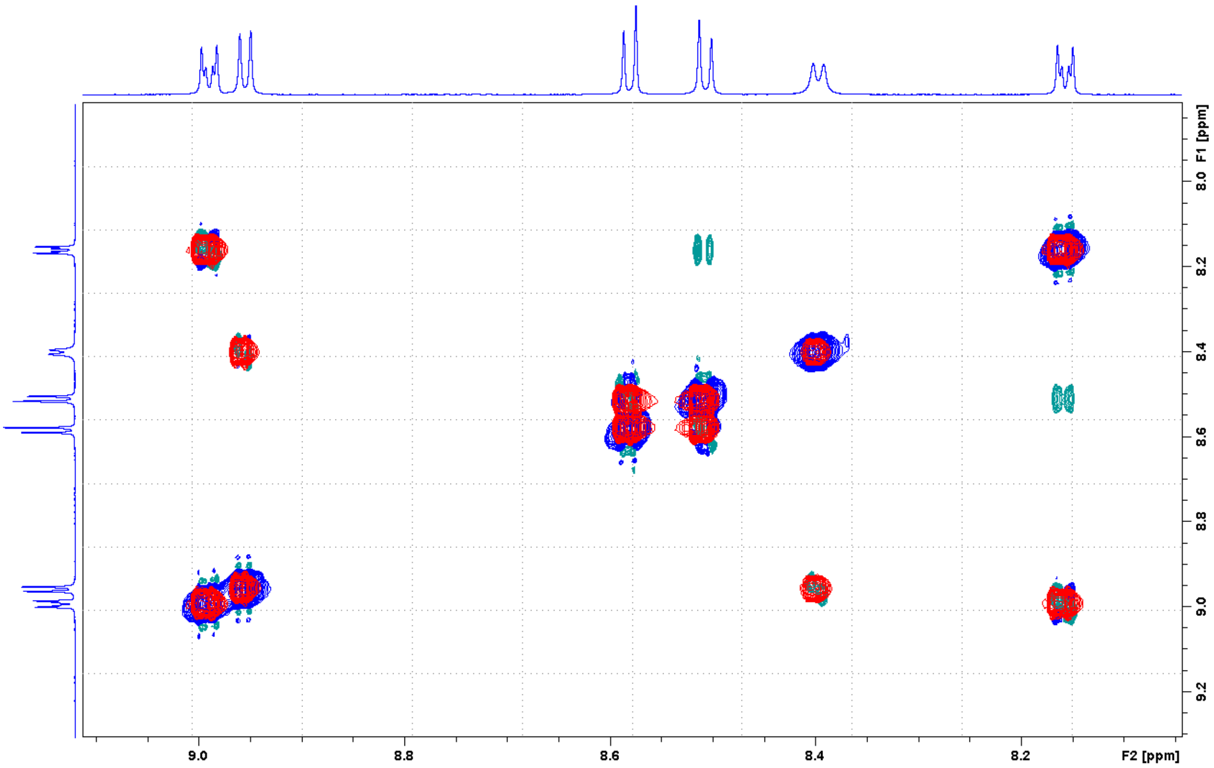

Figure S2. Overlapping of COSY and ROESY measurements of **py-CorM$_6$** in $CDCl_3$ at room temperature (focus on the β-pyrrolic signals).

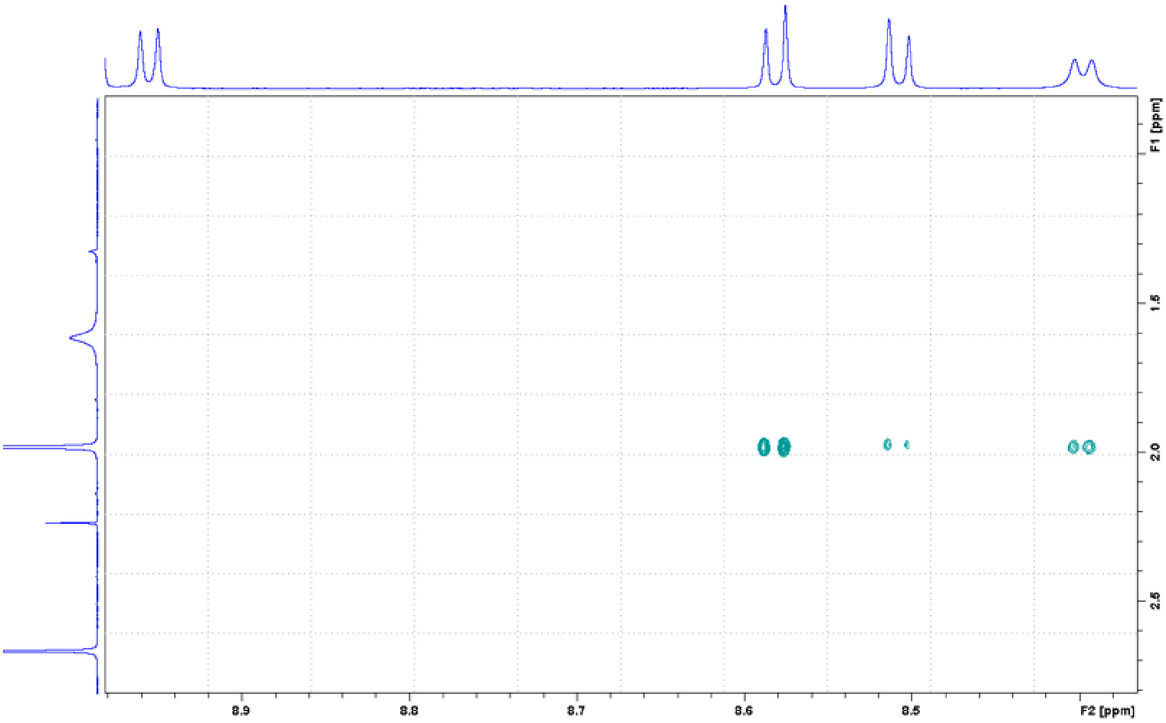

Figure S3. ROESY spectrum of **py-CorM$_6$** in $CDCl_3$ at room temperature (focus on the β-pyrrolic signals).

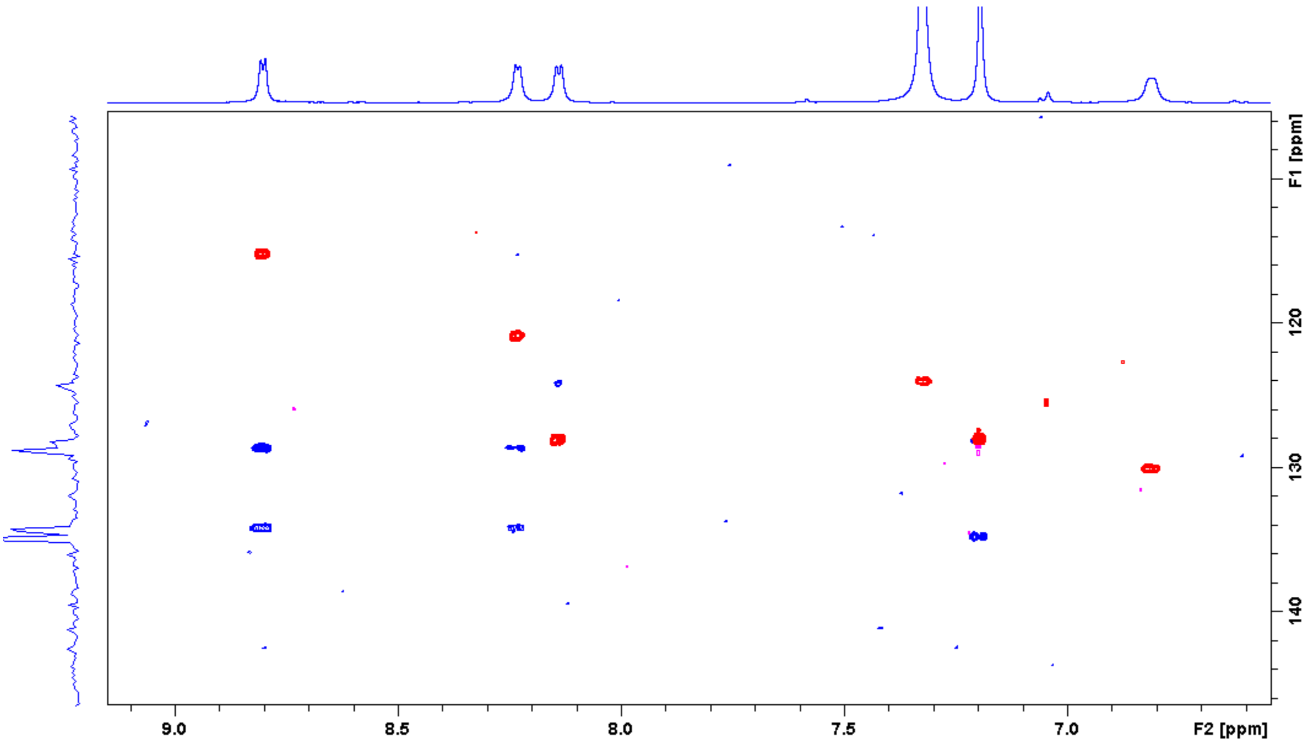

Figure S4. HSQC spectrum of **py-CorM$_6$•ZnPcR$_8$** in $CDCl_3$ at room temperature (focus on the β-pyrrolic signals).

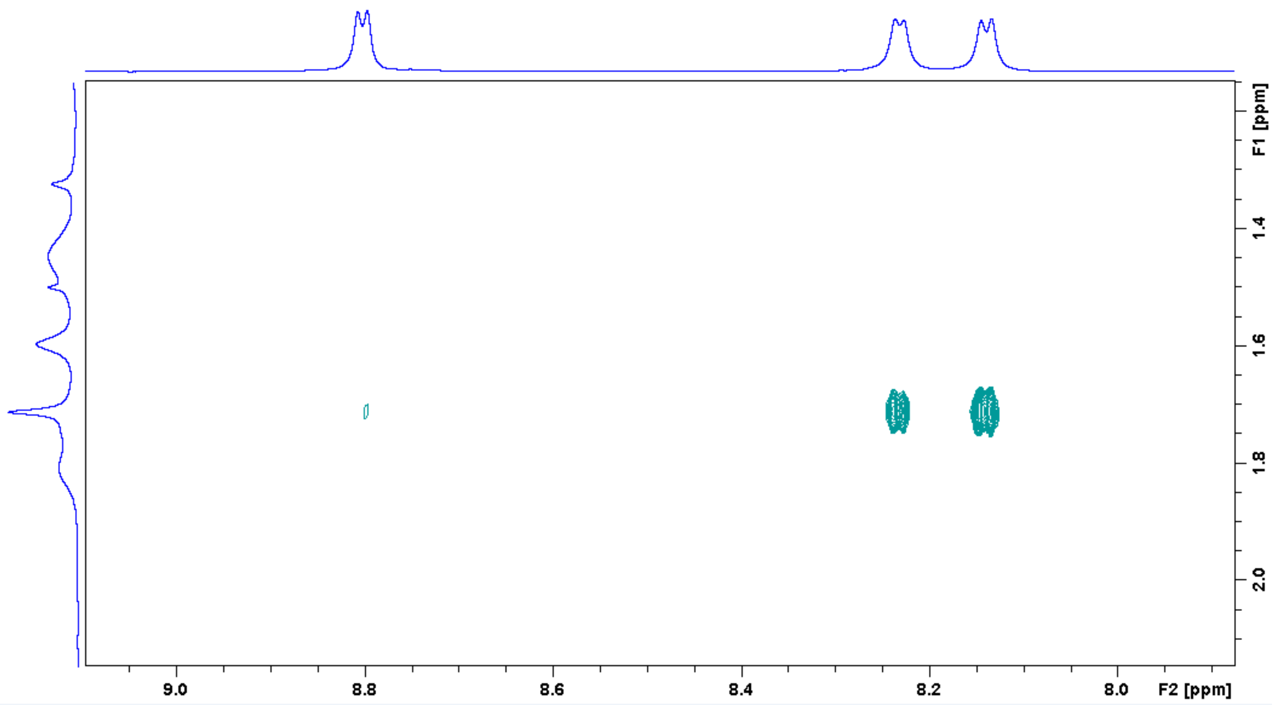

Figure S5. ROESY spectrum of **py-CorM$_6$•ZnPcR$_8$** in $CDCl_3$ at room temperature (focus on β-pyrrolic peaks).

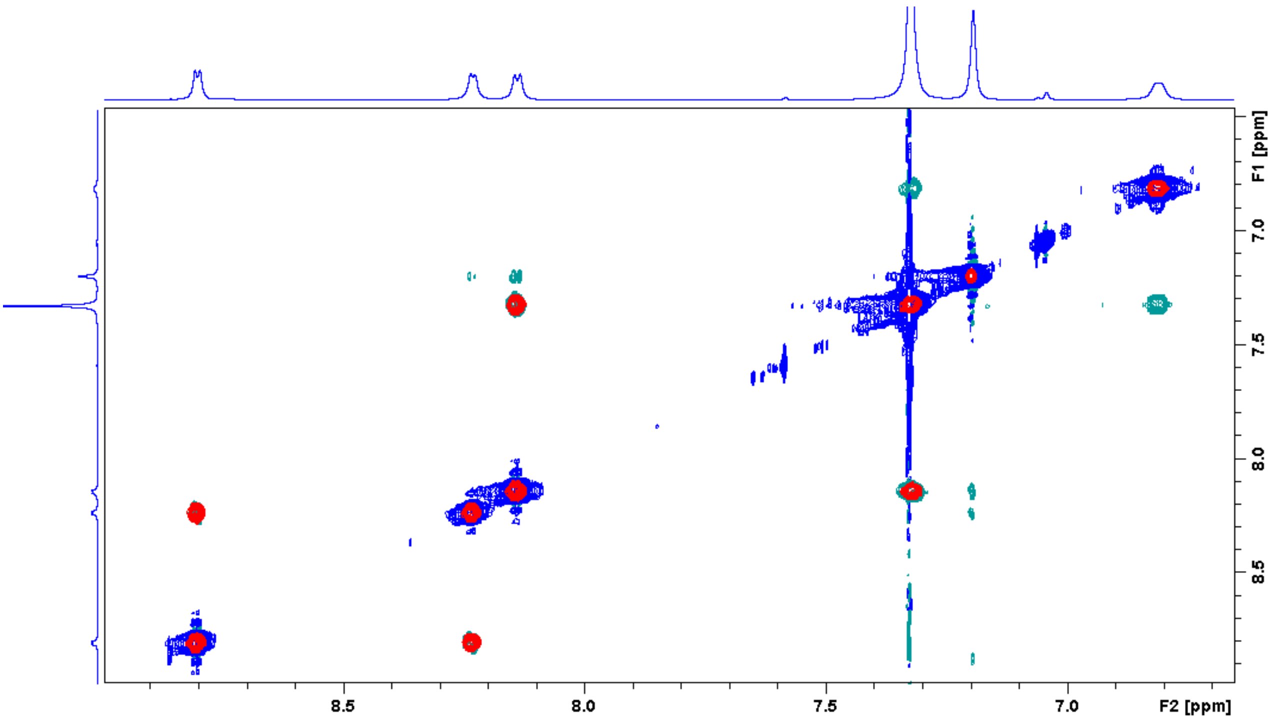

Figure S6. Overlapping of COSY and ROESY measurements of **py-CorM$_6$•ZnPcR$_8$** in $CDCl_3$ at room temperature (focus on β-pyrrolic peaks).

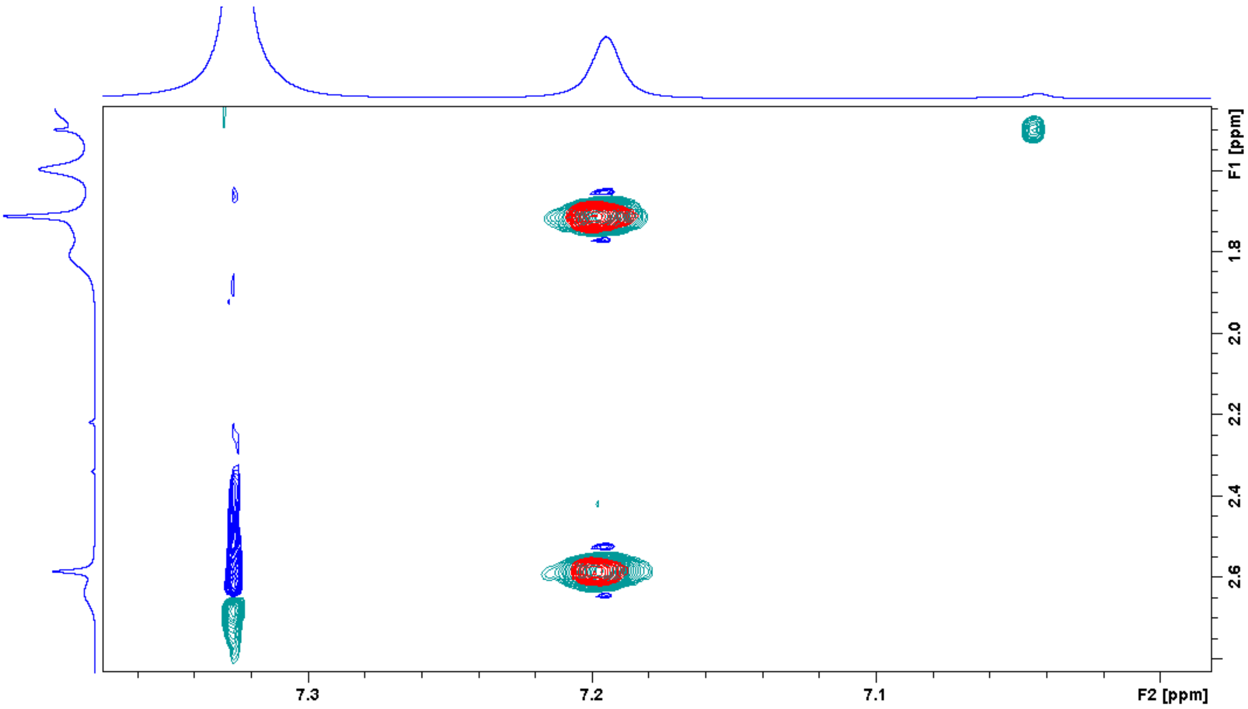

Figure S7. Overlapping of COSY and ROESY measurements of **py-CorM$_6$•ZnPcR$_8$** in $CDCl_3$ at room temperature (focus on aromatic mesityl peaks).

2. CYCLIC AND PULSE VOLTAMMETRY

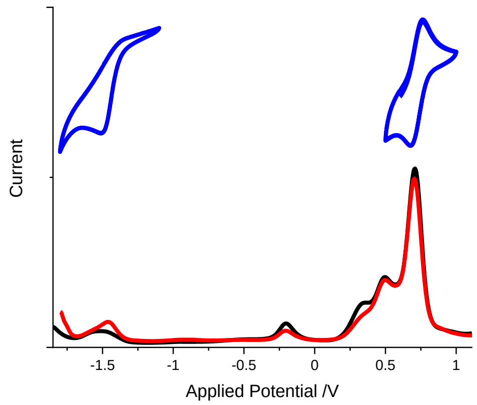

Figure S8. Square wave voltammograms (black: up-scan; red: down-scan; 0.01 V/s) of **py-CorM$_6$** in deaerated DCM (0.1 M TBAPF$_6$ as electrolyte) and corresponding cyclic-voltammograms (blue; 0.05 V/s).

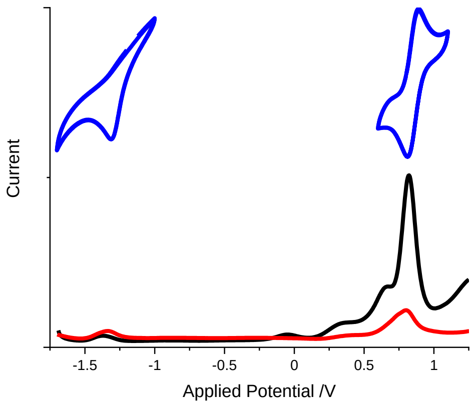

Figure S9. Square wave voltammograms (black: up-scan; red: down-scan; 0.01 V/s) of **py-CorF$_{10}$** in deaerated DCM (0.1 M TBAPF$_6$ as electrolyte) and corresponding cyclic-voltammograms (blue; 0.05 V/s).

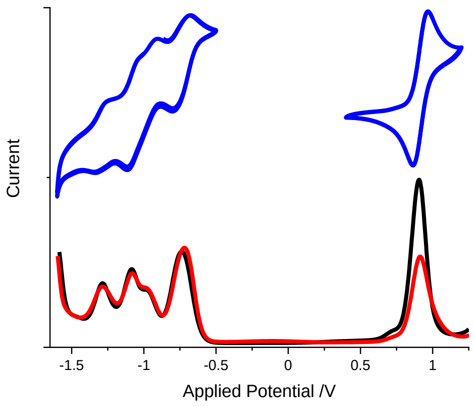

Figure S10. Square wave voltammograms (black: up-scan; red: down-scan; 0.01 V/s) of **$ZnPcR_8$** in deaerated DCM (0.1 M $TBAPF_6$ as electrolyte) and corresponding cyclic-voltammograms (blue; 0.05 V/s).

3. PHOTOPHYSICAL MEASUREMENTS

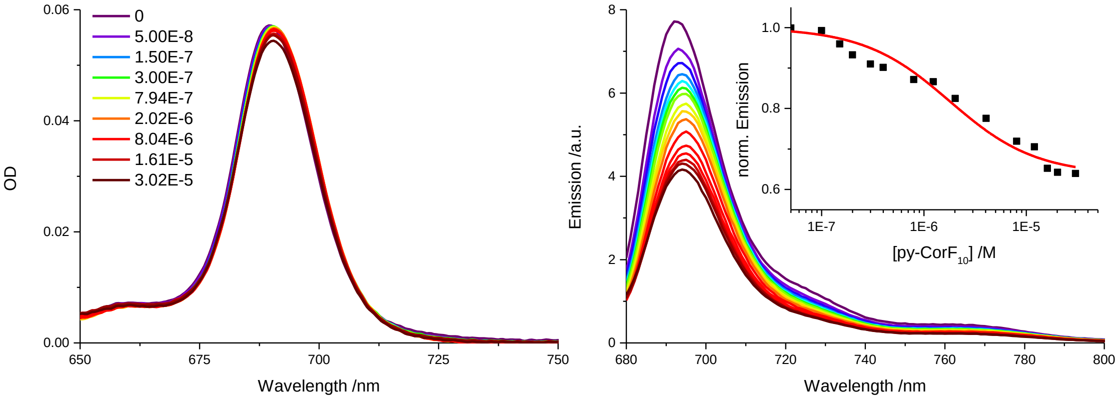

Figure S11. Absorption spectra on the left and fluorescence spectra on the right of **ZnPcR$_8$** (2 x 10^{-7} M) upon addition of variable **py-CorF$_{10}$** (0 - 3 x 10^{-5} M) in toluene at room-temperature. Inset on the right displays the normalized **ZnPcR$_8$** fluorescence to determine the binding constant.

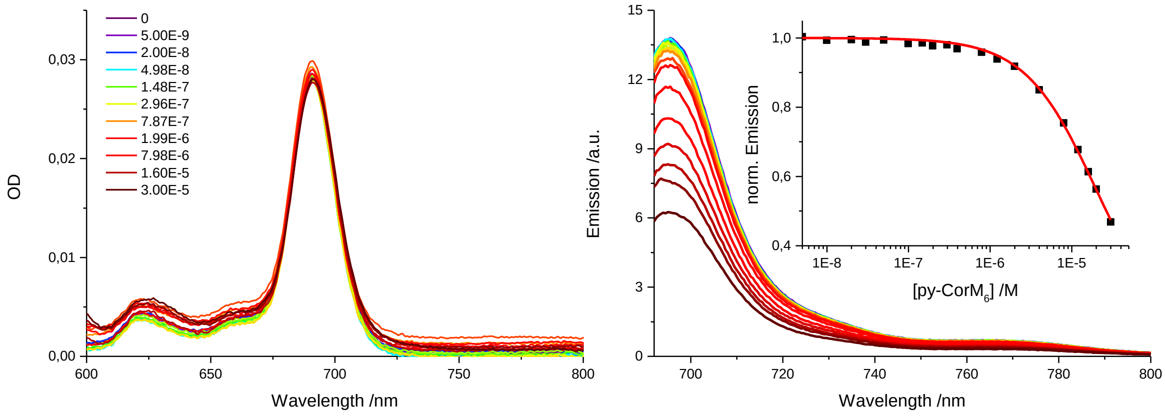

Figure S12. Absorption spectra on the left and fluorescence spectra on the right of **ZnPcR$_8$** (1 x 10^{-7} M) upon addition of variable **py-CorM$_6$** (0 - 3 x 10^{-5} M) in anisole at room-temperature. Inset on the right displays the normalized **ZnPcR$_8$** fluorescence to determine the binding constant.

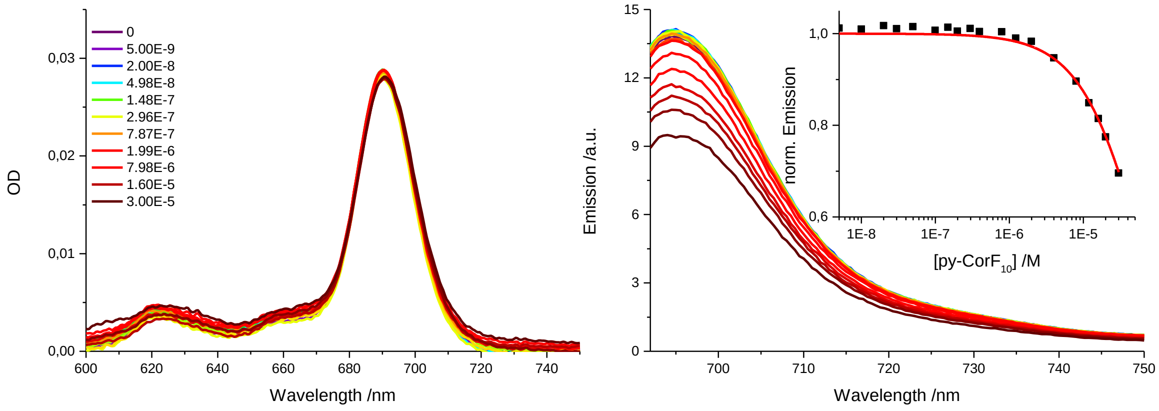

Figure S13. Absorption spectra on the left and fluorescence spectra on the right of **ZnPcR$_8$** (1×10^{-7} M) upon addition of variable **py-CorF$_{10}$** ($0 - 3 \times 10^{-5}$ M) in anisole at room-temperature. Inset on the right displays the normalized **ZnPcR$_8$** fluorescence to determine the binding constant.

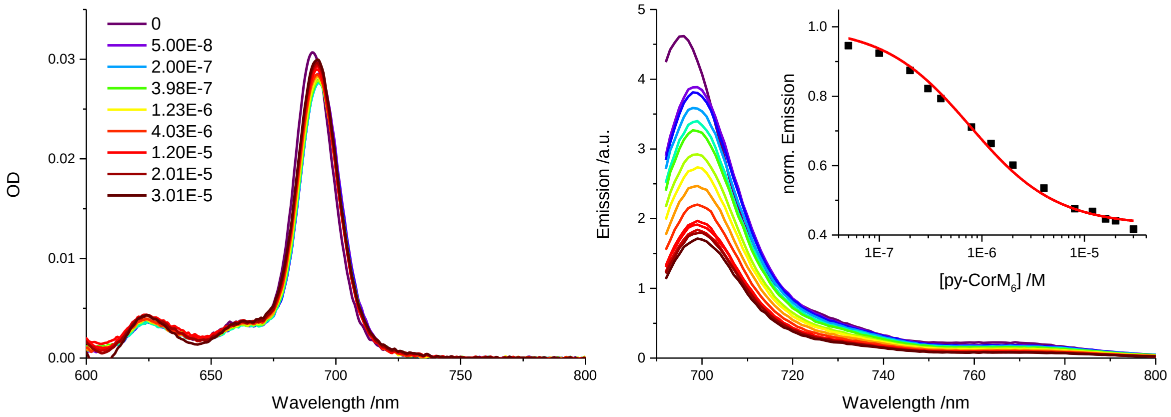

Figure S14. Absorption spectra on the left and fluorescence spectra on the right of **ZnPcR₈** (1 x 10^{-7} M) upon addition of variable **py-CorM₆** (0 - 3 x 10^{-5} M) in chlorobenzene at room-temperature. Inset on the right displays the normalized **ZnPcR₈** fluorescence to determine the binding constant.

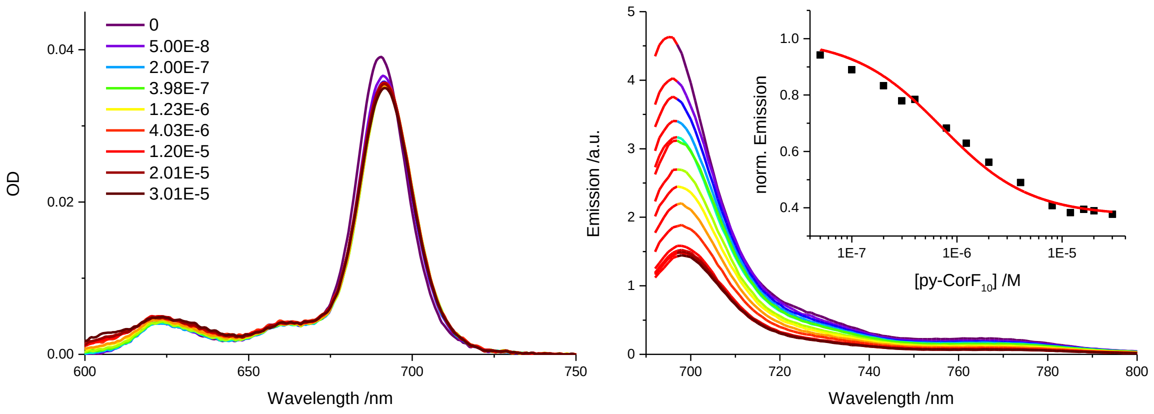

Figure S15. Absorption spectra on the left and fluorescence spectra on the right of **ZnPcR$_8$** (1 x 10^{-7} M) upon addition of variable **py-CorF$_{10}$** (0 - 3 x 10^{-5} M) in chlorobenzene at room-temperature. Inset on the right displays the normalized **ZnPcR$_8$** fluorescence to determine the binding constant.

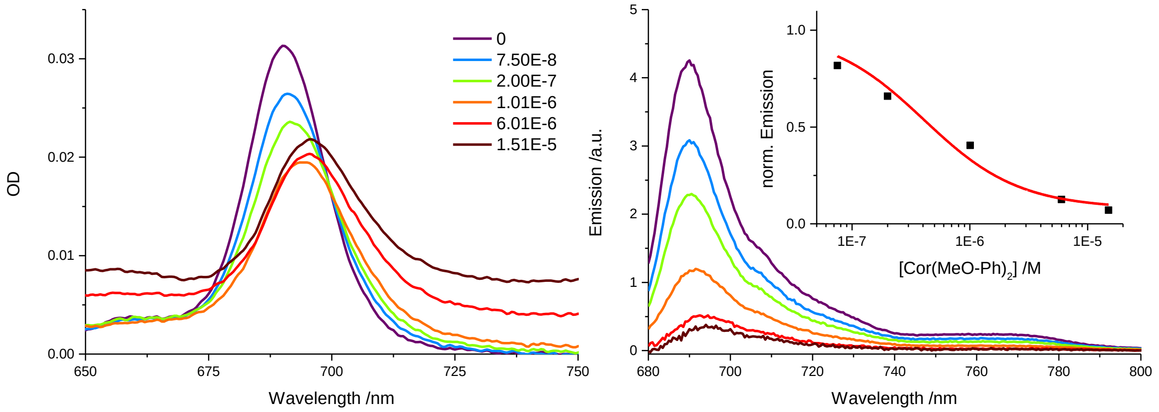

Figure S16. Absorption spectra on the left and fluorescence spectra on the right of **ZnPcR₈** (1 x 10^{-7} M) upon addition of variable **Cor**-reference (0 – 1.5 x 10^{-5} M) in toluene at room-temperature. Inset on the right displays the normalized **ZnPcR₈** fluorescence to determine the binding constant.

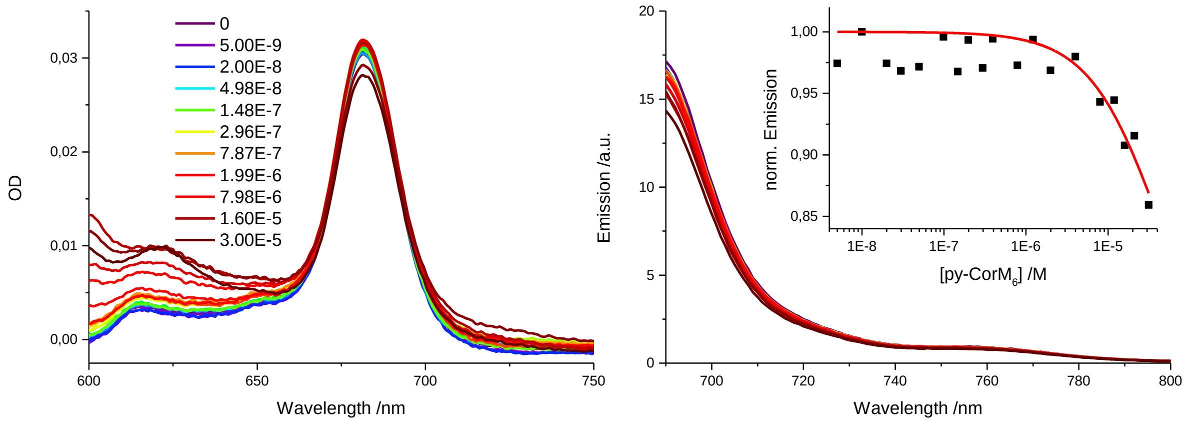

Figure S17. Absorption spectra on the left and fluorescence spectra on the right of **ZnPcR$_8$** (1 x 10^{-7} M) upon addition of variable **py-CorM$_6$** (0 - 3 x 10^{-5} M) in ethanol at room-temperature. Inset on the right displays the normalized **ZnPcR$_8$** fluorescence to determine the binding constant.

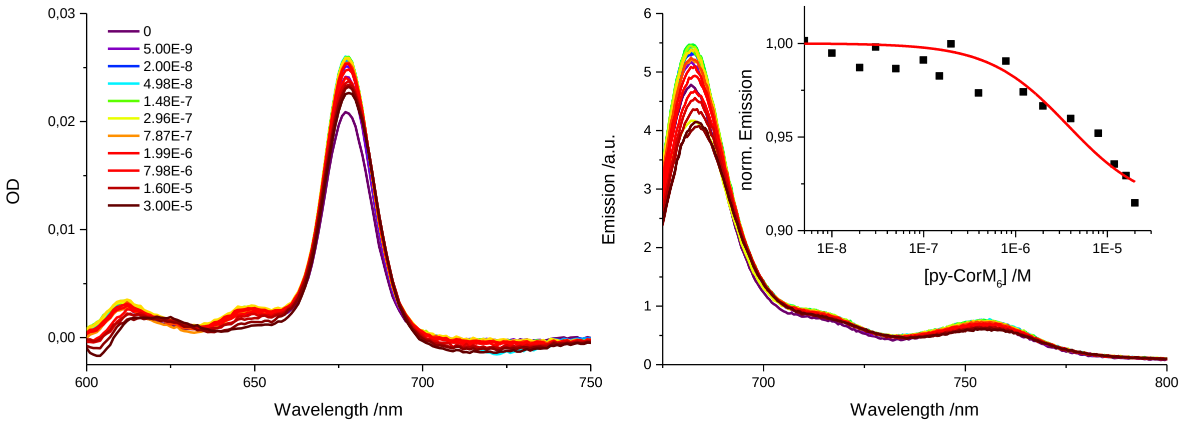

Figure S18. Absorption spectra on the left and fluorescence spectra on the right of ZnTtBuPc (1 x 10^{-7} M) upon addition of variable **py-CorM$_6$** (0 - 3 x 10^{-5} M) in toluene at room-temperature. Inset on the right displays the normalized ZnTtBuPc fluorescence to determine the binding constant.

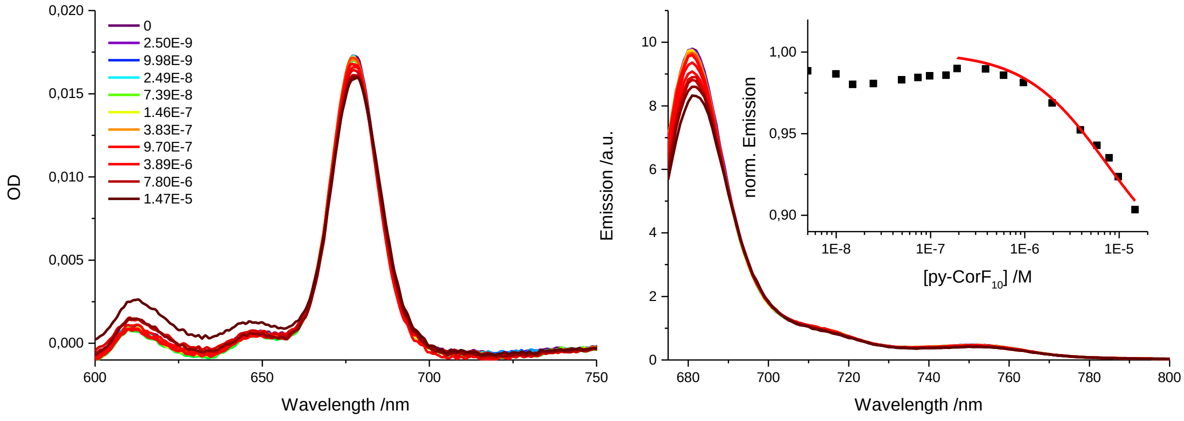

Figure S19. Absorption spectra on the left and fluorescence spectra on the right of ZnTtBuPc (5 x 10^{-8} M) upon addition of variable **py-CorF$_{10}$** (0 – 1.5 x 10^{-5} M) in toluene at room-temperature. Inset on the right displays the normalized ZnTtBuPc fluorescence to determine the binding constant.

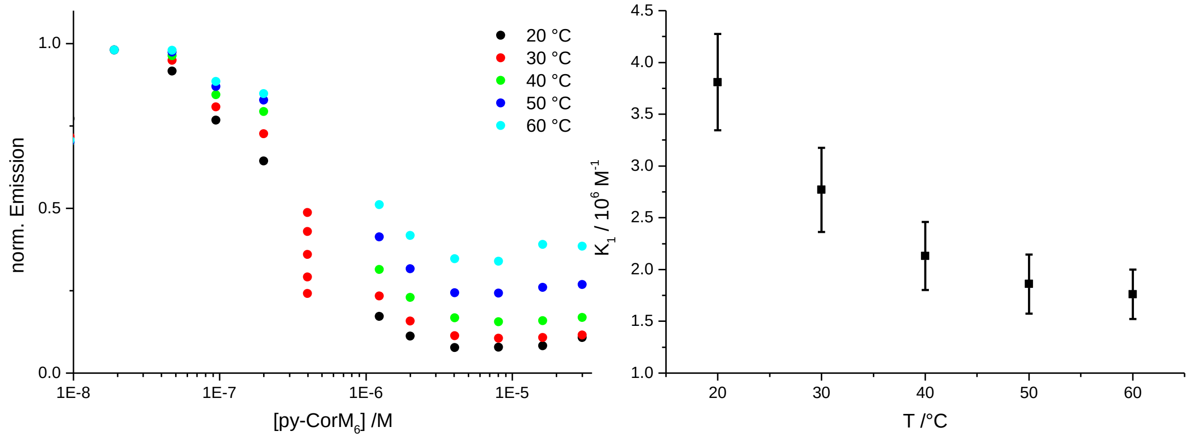

Figure S20. Normalized fluorescence on the left and association constants on the right acquired via 1:1 fitting of **ZnPcR₈** (10^{-7} M) upon addition of **py-CorM₆** in toluene at various temperatures.

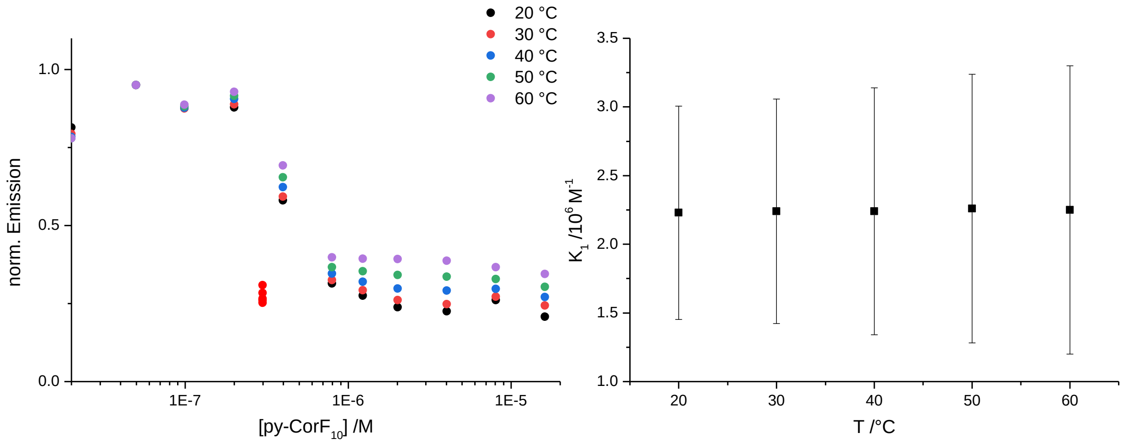

Figure S21. Normalized fluorescence on the left and association constants on the right acquired via 1:1 fitting of **ZnPcR$_8$** (10^{-7} M) upon addition of **py-CorF$_{10}$** in toluene at various temperatures.

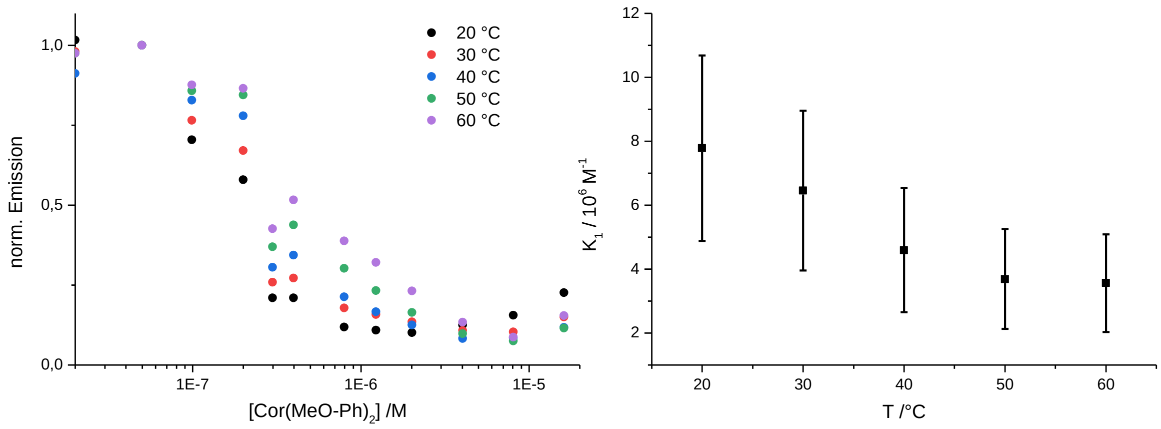

Figure S22. Normalized fluorescence on the left and association constants on the right acquired via 1:1 fitting of **ZnPcR**$_8$ (10^{-7} M) upon addition of a **Cor**-reference without a pyridyl anchor in toluene at various temperatures.

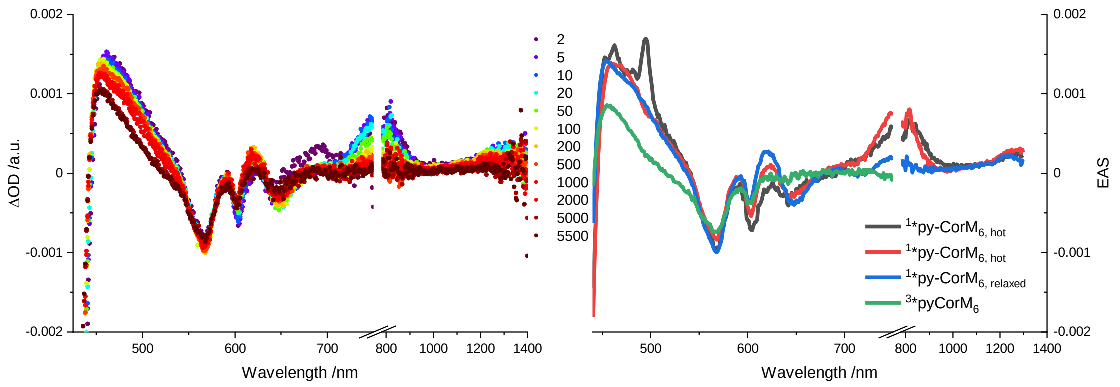

Figure S23. Differential absorption spectra on the left with time delays between 2 and 5500 ps of **py-CorM$_6$** obtained upon femtosecond flash-photolysis (excitation at 430 nm) in de-aerated toluene at room-temperature, evolution associated spectra of **^{1}*py-CorM$_{6, hot}$** (black, red), **^{1}*py-CorM$_{6, relaxed}$** (blue), and **^{3}*py-CorM$_6$** (green) on the right**.**

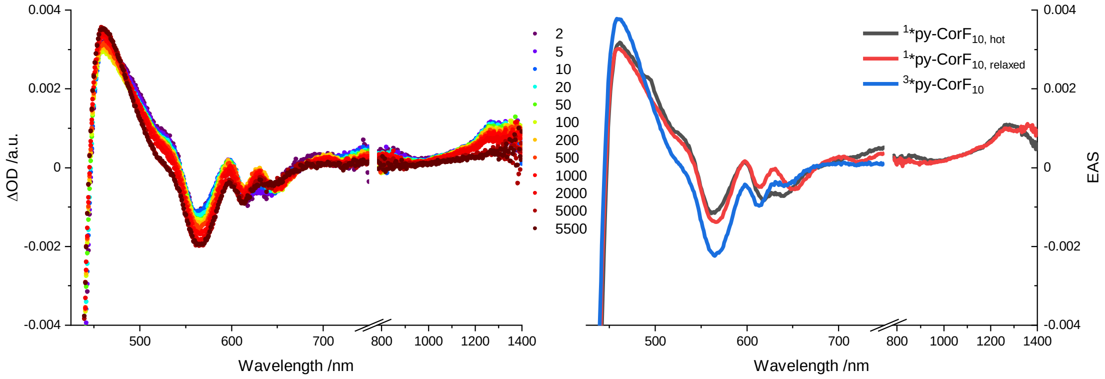

Figure S24. Differential absorption spectra on the left with delays between 2 and 5500 ps of **py-CorF$_{10}$** obtained upon femtosecond flash-photolysis (excitation at 430 nm) in de-aerated toluene at room-temperature, evolution associated spectra of **^{1}*py-CorF$_{10, hot}$** (black), **^{1}*py-CorF$_{10, relaxed}$** (red), and **^{3}*py-CorF$_{10}$** (blue) on the right.

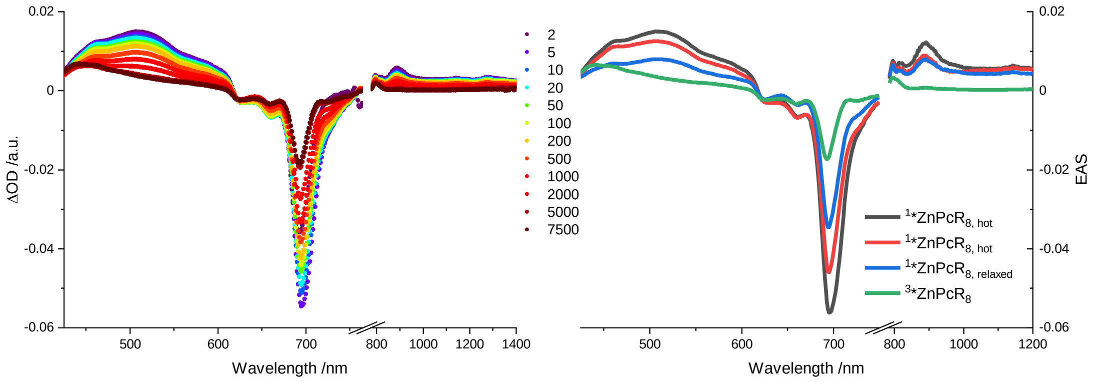

Figure S25. Differential absorption spectra on the left with delays between 2 and 7500 ps of **$ZnPcR_8$** obtained upon femtosecond flash-photolysis (excitation at 676 nm) in de-aerated toluene at room-temperature, evolution associated spectra of **$^{1*}ZnPcR_{8, hot}$** (black, red), **$^{1*}ZnPcR_{8, relaxed}$** (blue), and **$^{3*}ZnPcR_8$** (green) on the right.

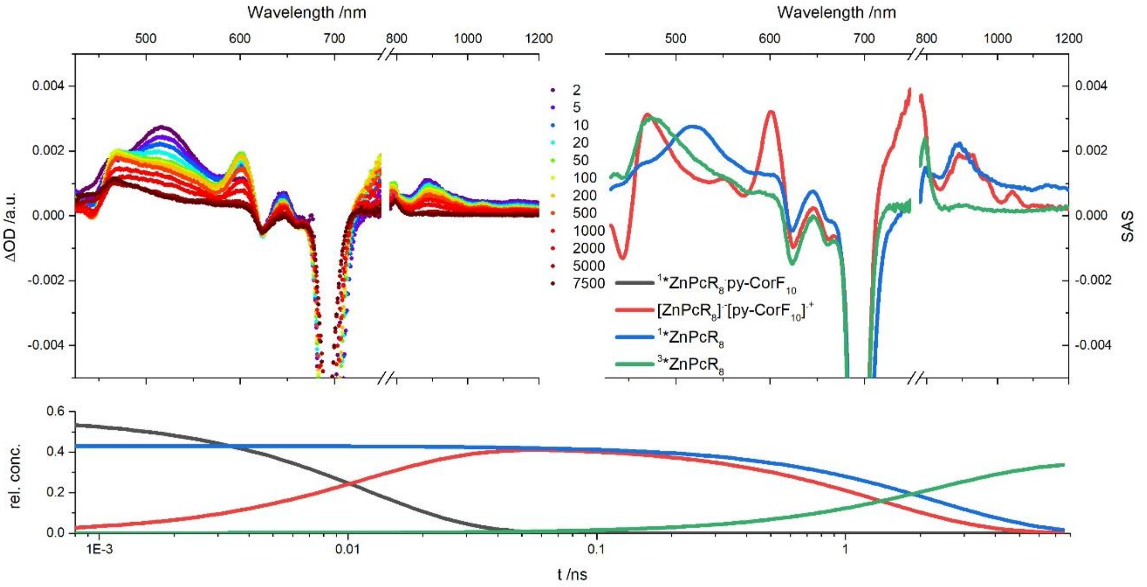

Figure S26. Differential absorption spectra on the top left with delays between 2 and 7500 ps, species associated spectra of **1*ZnPcR$_8$•py-CorF$_{10}$** (black), **[ZnPcR$_8$]$^{•-}$•[py-CorF$_{10}$]$^{•+}$** (red), **1*ZnPcR$_8$ (blue**), and **3*ZnPcR$_8$** (green) on the top right, and relative concentration profiles on the bottom of transient species of a 10:1 mixture of **py-CorF$_{10}$** and **ZnPcR$_8$** obtained upon femtosecond flash-photolysis (excitation at 676 nm) in de-aerated toluene at room-temperature.

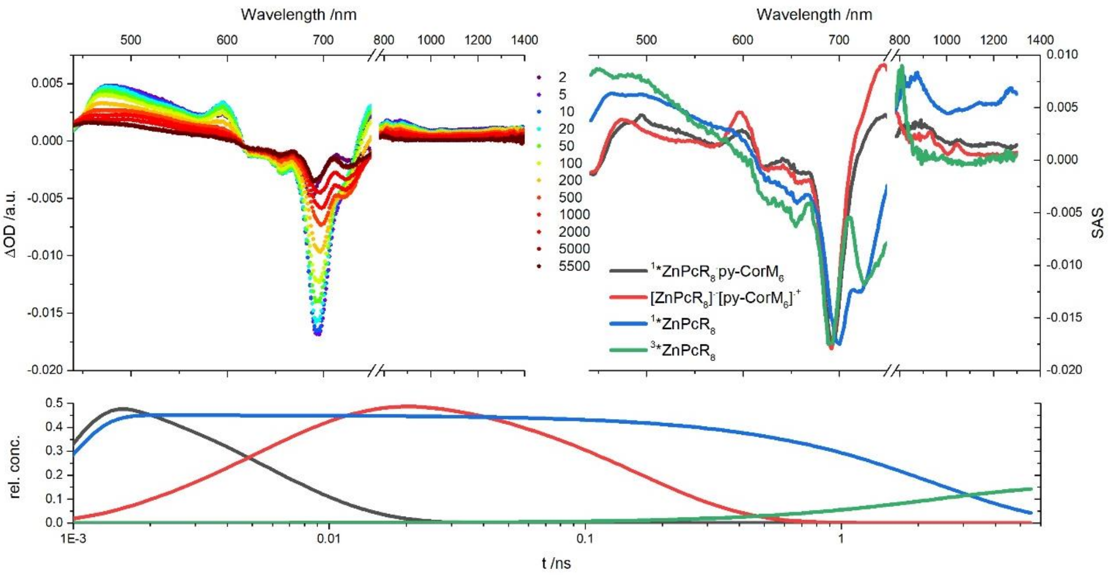

Figure S27. Differential absorption spectra on the top left with delays between 2 and 5500 ps, species associated spectra of **^{1}*ZnPcR$_8$•py-CorM$_6$** (black), **[ZnPcR$_8$]$^{•-}$•[py-CorM$_6$]$^{•+}$** (red), **^{1}*ZnPcR$_8$ (blue**), and **^{3}*ZnPcR$_8$** (green) on the top right, and relative concentration profiles on the bottom of transient species of a 1:5 mixture of **py-CorM$_6$** and **ZnPcR$_8$** obtained upon femtosecond flash-photolysis (excitation at 430 nm) in de-aerated toluene at room-temperature.

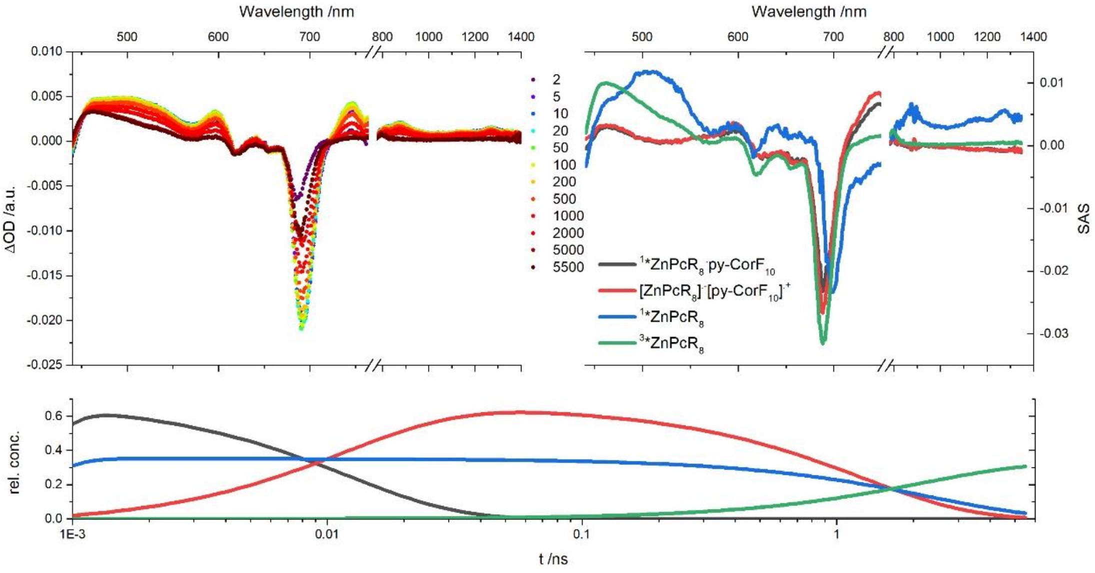

Figure S28. Differential absorption spectra on the top left with delays between 2 and 5500 ps, species associated spectra of **^{1}*ZnPcR$_8$•py-CorF$_{10}$** (black), **[ZnPcR$_8$]$^{•-}$•[py-CorF$_{10}$]$^{•+}$** (red), **^{1}*ZnPcR$_8$ (blue**), and **^{3}*ZnPcR$_8$** (green) on the top right, and relative concentration profiles on the bottom of transient species of a 1:5 mixture of **py-CorF$_{10}$** and **ZnPcR$_8$** obtained upon femtosecond flash-photolysis (excitation at 430 nm) in de-aerated toluene at room-temperature.

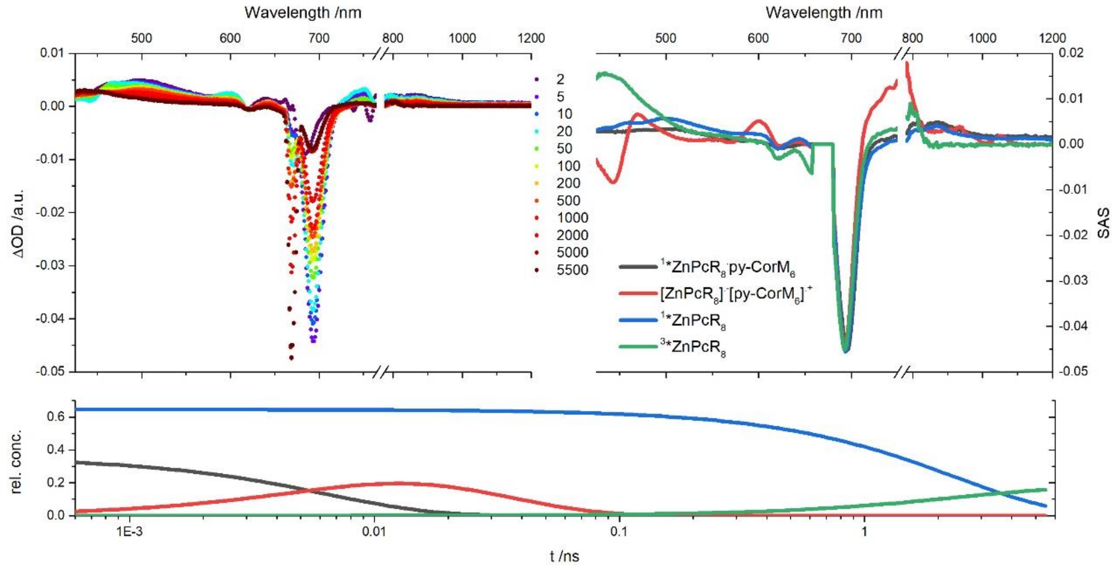

Figure S29. Differential absorption spectra on the top left with delays between 2 and 5500 ps, species associated spectra of **^{1}*ZnPcR$_8$•py-CorM$_6$** (black), **[ZnPcR$_8$]$^{•-}$•[py-CorM$_6$]$^{•+}$** (red), **^{1}*ZnPcR$_8$ (blue**), and **^{3}*ZnPcR$_8$** (green) on the top right, and relative concentration profiles on the bottom of transient species of a 5:1 mixture of **py-CorM$_6$** and **ZnPcR$_8$** obtained upon femtosecond flash-photolysis (excitation at 670 nm) in de-aerated anisole at room-temperature.

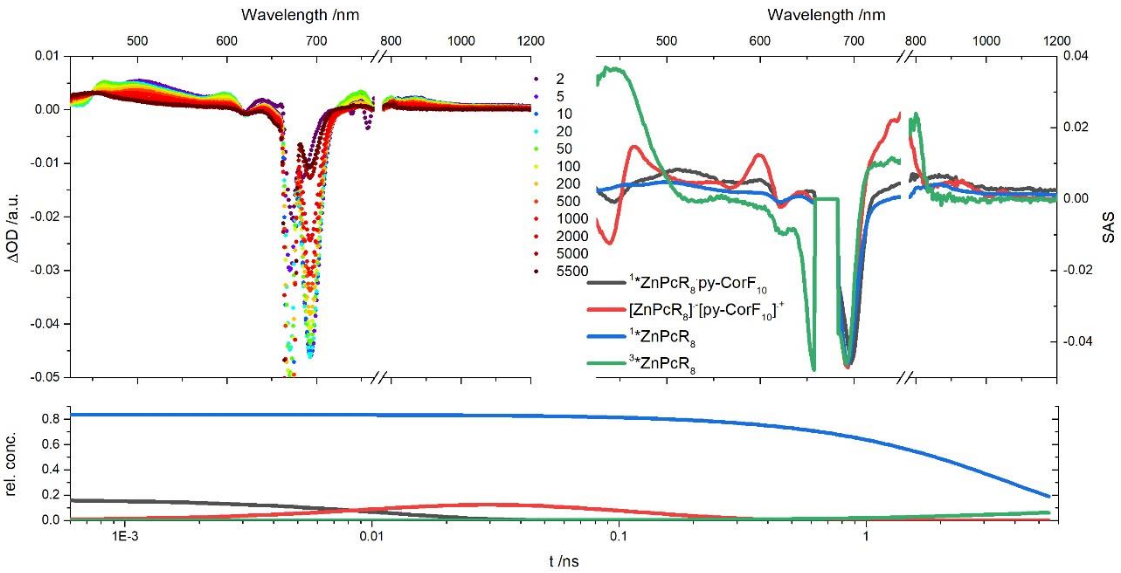

Figure S30. Differential absorption spectra on the top left with time delays between 2 and 5500 ps, species associated spectra of **^{1*}ZnPcR$_8$•py-CorF$_{10}$** (black)**, [ZnPcR$_8$]$^{•-}$•[py-CorF$_{10}$]$^{•+}$** (red), **^{1*}ZnPcR$_8$** (blue), and ^{3*}**ZnPcR$_8$** (green) on the top right, and relative concentration profiles on the bottom of transient species of a 5:1 mixture of **py-CorF$_{10}$** and **ZnPcR$_8$** obtained upon femtosecond flash-photolysis (excitation at 670 nm) in de-aerated anisole at room-temperature.
